# High-throughput sequencing application in the detection and discovery of viruses associated with the regulated citrus leprosis disease complex

**DOI:** 10.3389/fpls.2022.1058847

**Published:** 2023-01-24

**Authors:** Chellappan Padmanabhan, Schyler Nunziata, Guillermo Leon M., Yazmín Rivera, Vessela A. Mavrodieva, Mark K. Nakhla, Avijit Roy

**Affiliations:** ^1^ United States Department of Agriculture (USDA), Animal Plant Health Inspection Service, Plant Protection and Quarantine, Science and Technology, Plant Pathogen Confirmatory Diagnostics Laboratory, Laurel, MD, United States; ^2^ AGROSAVIA, Centro de Investigación La Libertad, Meta, Colombia; ^3^ United States Department of Agriculture (USDA), Agricultural Research Service, Molecular Plant Pathology Laboratory, Beltsville Agricultural Research Center, Beltsville, MD, United States

**Keywords:** Brevipalpus transmitted viruses, Citrus leprosis virus, *Cilevirus*, *Dichorhavirus*, High-throughput sequencing, *Kitaviridae*, Mixed infection, Real time PCR

## Abstract

Citrus leprosis (CiL) is one of the destructive emerging viral diseases of citrus in the Americas. Leprosis syndrome is associated with two taxonomically distinct groups of *Brevipalpus*-transmitted viruses (BTVs), that consist of positive-sense *Cilevirus*, *Higrevirus*, and negative-sense *Dichorhavirus*. The localized CiL symptoms observed in multiple citrus species and other alternate hosts indicates that these viruses might have originated from the mites and eventually adopted citrus as a secondary host. Genetic diversity in the genomes of viruses associated with the CiL disease complex have complicated current detection and diagnostic measures that prompted the application of High-Throughput Sequencing (HTS) protocols for improved detection and diagnosis. Two cileviruses are known to infect citrus, and among them only citrus leprosis virus C2 (CiLV-C2) hibiscus strain (CiLV-C2H) has been reported in hibiscus and passion fruit in the US. Based on our current CiL disease complex hypothesis, there is a high probability that CiL disease is associated with more viruses/strains that have not yet been identified but exist in nature. To protect the citrus industry, a Ribo-Zero HTS protocol was utilized for detection of cileviruses infecting three different hosts: *Citrus* spp., *Swinglea glutinosa*, and *Hibiscus rosa-sinensis.* Real-time RT-PCR assays were used to identify plants infected with CiLV-C2 or CiLV-C2H or both in mixed infection in all the above-mentioned plant genera. These results were further confirmed by bioinformatic analysis using HTS generated data. In this study, we utilized HTS assay in confirmatory diagnostics to screen BTVs infecting *Dieffenbachia* sp. (family: Araceae), *Passiflora edulis* (Passifloraceae), and *Smilax auriculata* (Smilacaceae). Through the implementation of HTS and downstream data analysis, we detected not only the known cileviruses in the studied hosts but also discovered a new strain of CiLV-C2 in hibiscus from Colombia. Phylogenetically, the new hibiscus strain is more closely related to CiLV-C2 than the known hibiscus strain, CiLV-C2H. We propose this strain to be named as CiLV-C2 hibiscus strain 2 (CiLV-C2H2). The findings from the study are critical for citrus growers, industry, regulators, and researchers. The possible movement of CiLV-C2H2 from hibiscus to citrus by the *Brevipalpus* spp. warrants further investigation.

## Introduction

The United States is the fifth largest citrus producer in the world. California and Florida accounted for 98% of total US citrus production (62% and 36%, respectively) whereas Texas and Arizona contributed the remaining 2%. In 2021-2022, production (5.61 million tons) decreased by 19% compared to the 2020-21 season (Citrus Fruits August 2022 Summary, USDA, National Agricultural Statistics Service). Huanglongbing disease has reduced citrus growing areas in Florida by nearly 40% and production has been reduced by two thirds compared to 20 years ago. Citrus yields will be reduced further if the reemergence of citrus leprosis (CiL) in U.S. mainland occurs. Citrus leprosis was first reported from Florida more than 125 years ago and was originally named scaly bark due to merged local lesions on green bark (Fawcett, 1911). Degradome sequencing of herbarium sample from Florida confirmed the association of CiLV-N0, a distant relative of *Dichorhavirus orchidaceae* [orchid fleck virus, (OFV)], with CiL disease symptoms (Hartung et al., 2015). Even though this virus is not known to exist anywhere in the world today, other related strains of OFV have been reported recently from Florida on ornamental hosts (Fife et al., 2021; Dey et al., 2022). In addition, a strain of citrus leprosis virus C2 (*Cilevirus*) was reported in Hibiscus in Tampa, FL, USA in 2017 (Roy et al., 2018a; Roy et al., 2018c) and since then the virus has been found in several counties in Florida.

Citrus leprosis disease (CiLD) is a major economic problem for the South American citrus industry. In the last decade, CiL viruses (CiLVs) have spread swiftly from South to Central America and were reported from Mexico. CiLD is the most significant virus disease affecting citrus in Brazil, where up to US $54 million per year was expended to manage the disease (Bassanezi et al., 2019). CiLD is associated with two unrelated taxa of single-stranded RNA viruses; one taxon includes the cytoplasmic, positive sense *Cilevirus* and *Higrevirus* that replicate in the cytoplasm. The other taxon is the nuclear, negative sense *Dichorhavirus* that replicates in or near the nucleus. CiLD is a classic example of convergent evolution as both virus genera produce similar symptoms on susceptible citrus species and are transmitted by flat mite vectors *Brevipalpus* spp., which are present in Florida and other citrus growing regions in the US. Re-introduction of CiLD can be prevented if the Brevipalpus population is controlled by acaricide application along with other management practices (Argolo et al., 2020).

*Cilevirus leprosis* [citrus leprosis virus-C (CiLV-C)] (Locali-Fabris et al., 2006; Ramos-González et al., 2016) and *Cilevirus colombiaense* [citrus leprosis virus-C2 (CiLV-C2)] (Roy et al., 2013b) belong to the genus *Cilevirus*, family *Kitaviridae*, and are considered the most devastating viruses infecting citrus in Brazil. Three distinct strains of CiLV-C (CRD, SJP and ASU) and two distinct strains of CiLV-C2 (citrus and hibiscus) have been reported. On the other hand, there are two sub-groups of *Dichorhavirus*. The first sub-group of dichorhaviruses include citrus leprosis virus N (CiLV-N) and citrus chlorotic spot virus (CiCSV) those produce citrus leprosis like symptoms and is restricted to Brazil (Ramos-González et al., 2017; Chabi-Jesus et al., 2018). The second sub-group of dichorhaviruses cause citrus leprosis in Mexico, Colombia and South Africa and share remarkable similarity in gene order, number and length of the open reading frames (ORFs), and sequence with OFV. The OFV includes two orchid strains (OFV-Orc1 and OFV-Orc2) (Kondo et al., 2006; Kondo et al., 2017) and two citrus strains (OFV-Cit1 and OFV-Cit2) (Roy et al., 2015b; Roy et al., 2020).

Both positive and negative sense *Cilevirus* and *Dichorhavirus* genome, respectively, are comprised of two RNA segments. The *Cilevirus* RNA1 genome segment consists of two ORFs, is approximately 9.0 kb in length and encodes the RNA dependent RNA polymerase (RdRp) and the putative coat protein (p29) genes. The RNA2 genome segment is almost 5.0 kb in length and has four/five ORFs. Except the second ORF (p7 in CiLV-C2 and p13 in passion fruit green spot virus (PfGSV) the other four ORFs p15, p61, p32, and p24 are present among known *Cilevirus* species/strains (Roy et al, 2013b; Ramos-González et al., 2020). The genome of bipartite dichorhaviruses is approximately 12 kb in length and contains five non-overlapping ORFs of RNA1 and single ORF of RNA2 (Kondo et al., 2006; Roy et al, 2015; Kondo et al., 2017; Ramos-González et al., 2017; Roy et al, 2020).

In Colombia, *Swinglea glutinosa* was identified as the first non-citrus natural host of CiLV-C (León et al., 2008). CiLV-C also naturally infects *Commelina benghalensis* and can be experimentally transmitted to plants of 28 families (Nunes et al., 2012; Garita et al., 2014; Arena et al., 2017). Apart from Swinglea, CiLV-C2 has a natural host range limited to the Malvaceae (hibiscus) and Araceae (*Dieffenbachia* sp.) (Roy et al., 2015a). The citrus strain of CiLV-C2 was restricted to Colombia whereas the hibiscus strain of citrus leprosis virus 2 (CiLV-C2H) was reported in hibiscus from Hawaii and Florida (Melzer et al., 2013; Roy et al., 2018a) and in citrus in Colombia but in mixed infection with CiLV-C2 (Roy et al., 2018b). CiLV-C2H is the only *Cilevirus* reported at present in the US that might infect citrus and cause leprosis.

CiL causes local lesions, and necrotic, or chlorotic spots in leaves, twigs, and fruit. Lesions consist of necrotic centers surrounded by chlorotic halos, and often coalesce and form larger leaf spots. Advanced infection can lead to plant death (Bastianel et al., 2010). Diagnosis of citrus leprosis is principally by the observation of characteristic local lesions. Electron microscopy of leprosis-like symptomatic leaf samples reveal either short bacilliform virions (40 - 50 x 100-110nm) within cytoplasmic endoplasmic reticulum (Roy et al., 2013b) or bullet shaped virions (20-30 x 120-135 nm) in the nucleus (Roy et al., 2015b) which are characteristic of *Cilevirus* and *Dichorhavirus* infections, respectively. CiLV-C and CiLV-C2 antibodies were developed by our group for serological testing (Choudhary et al., 2013; Choudhary et al., 2014; Choudhary et al., 2017) but are not available commercially. Conventional reverse-transcriptase polymerase chain reaction (RT-PCR)-based diagnosis methods are available for rapid and reliable detection of cytoplasmic and nuclear CiLVs (Roy et al., 2020; Chabi-Jesus et al., 2021). However, these diagnostic tools are not always sufficient to detect and identify the 12 different viruses/strains currently reported in association with CiLD in the Americas and South Africa. Due to this diversity, the existing technology is often not able to detect new virus species or strains in symptomatic samples.

High-Throughput Sequencing (HTS) has become an important tool for the detection and discovery of plant viruses. In this study, we used HTS as an alternative diagnostic tool for the detection of citrus leprosis associated cile- and dichorha-viruses on various hosts. To initiate the current study, we selected *Cilevirus* positive and negative RNA extracts from *Citrus* spp., *Swinglea glutinosa*, and *Hibiscus rosa-sinensis.* Current HTS protocol was not only used for diagnosis of *Brevipalpus*-transmitted viruses (BTVs) associated with CiLD complex in several plant species but also used for the discovery of a new hibiscus strain of CiLV-C2 (CiLV-C2H2) in Colombia from a RNA extract that tested negative by other means.

## Materials and methods

### Source of citrus leprosis infected samples

Samples of different hosts showing symptoms of BTVs were collected from following citrus-producing regions in Colombia, including the Andean region (Boyacá, Cundinamarca, Santander, Tolima, and Cauca Valley Departments), central coffee-growing region (Caldas, Quindío, Risaralda Departments), and Orinoquia region at east plains (Casanare and Meta Departments). Multiple BTVs suspect samples from citrus, hibiscus, swinglea and dieffenbachia were collected and sent for testing to the Plant Pathogen Confirmatory Diagnostics Laboratory (PPCDL), United States. Furthermore, several BTV positive samples from Hawaii, and Florida were received for confirmatory diagnostics and current HTS method was utilized. These samples included: rough lemon (*C. jambhiri*), mandarin (*C. reticulata*) and passion fruit (*Passiflora edulis*) from Hawaii; and hibiscus and greenbrier (*Smilax auriculata*), from Florida. Samples were processed under biological safety cabinet and the symptomatic lesions were excised and chopped into small pieces. About ~100 mg of symptomatic tissue of hibiscus or 6 to 12 leprosis lesions of citrus or swinglea were transferred to 2 ml microcentrifuge tubes and stored at -80°C until processed for RNA isolation.

### RNA isolation

To avoid a false positive in the RT-PCR assays from infected Brevipalpus mites, total RNA was extracted from 100 mg of Brevipalpus-free leaf tissues. Two different RNA extraction methods were chosen to compare the quality of extracted RNA from the leaves of three different hosts (citrus, swinglea and hibiscus). All the tissues were ground with a mortar and pestle to a fine powder using liquid nitrogen following extraction with TRIzol according to manufacturer’s (ThermoFisher Scientific, USA) instruction. Extracted RNA was treated with 1 unit of RNase free DNase I per 1-5 µg of total RNA in a 50 µl total volume (Thermo Fisher Scientific, USA) and incubated for 20-30 minutes at room temperature (25-37°C). The DNase-I treated RNA was resuspended in nuclease free water and its concentration was measured with a NanoDrop spectrophotometer (ThermoFisher Scientific, USA). The DNA-free high-quality RNA was also confirmed in a TapeStation (Agilent 4200, Santa Clara, CA). The RNeasy Plant Mini Kit (Qiagen, USA) was also used for total RNA extraction. During this process, 100 mg tissue with lesions was homogenized using a FastPrep FP-24 instrument (MP Biomedical). RNA concentrations were measured with Qubit fluorometer (Invitrogen, Carlsbad, CA), and the quality was tested using TapeStation (Agilent, Santa Clara, CA) as per manufacturer’s instructions.

### Real-time RT-PCR

Real time RT-PCR assays were performed using previously published *Cilevirus* species/strain (CiLV-C, CiLV-C2 and CiLV-C2H) specific primers and TaqMan probes (IDT and ThermoFisher Scientific, USA) (Choudhary et al., 2015; Adducci et al., 2018). The 20 µL reaction was setup using 5 µL of 4X TaqMan Fast Virus 1-Step Master Mix, 2.4 µL of primer probe mixes (consisted of virus specific primers 0.96 µM, probe 0.48 µM, Nad5 internal control primer 0.24 µM, probe 0.12 µM), 2 µL of RNA samples (~50 ng/µL) and 10.6 µL of molecular grade water and then run on a QuantStudio5 Real-Time PCR machine (ThermoFisher Scientific, USA). The following thermal cycling conditions were utilized: Hold stage- instrument automatic ramp 2.552°C/s, 50 °C for 10 min, denaturation at 95 °C for 20 sec, PCR stage-instrument automatic ramp 1.988°C/s between cycling temperature, 40 cycles of 95 °C for 3 sec and 62 °C for 30 sec.

### Ribo-Zero library preparation and high- throughput sequencing

**Figure 1:**
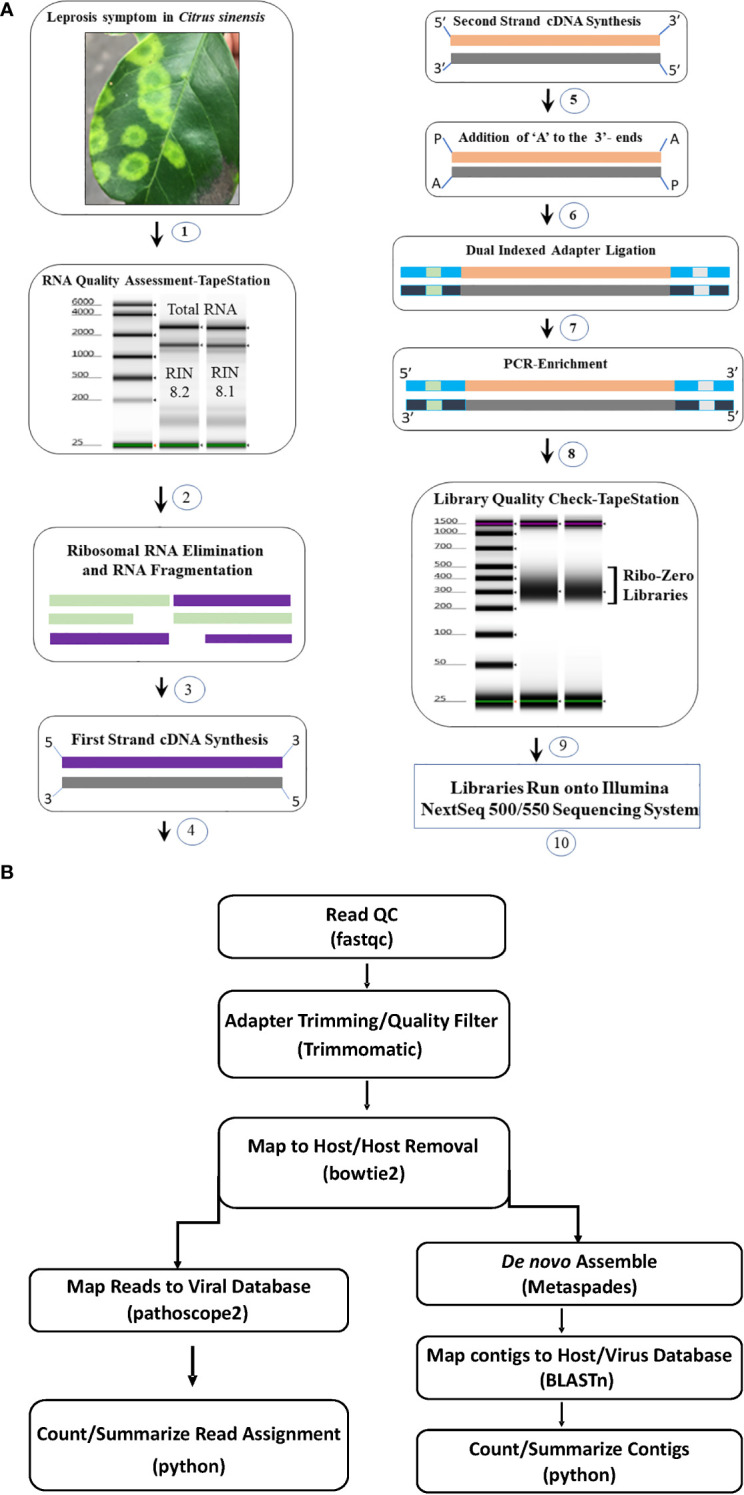
**(A)** Total RNA Ribo depleted High Throughput Sequencing (HTS) library preparation work flow used for detection of viruses associated with citrus leprosis disease complex. (1) Selection of citrus leprosis disease sample based on symptoms, (2) Total RNA extraction and quality check using TapeStation, (3) Preparation of Ribo-Zero library: rRNA removal and fragmentation, (4) first strand cDNA synthesis (5) second strand cDNA synthesis (6) Adenylation, (7) Adapter ligation, (8) Library enrichment and (9) Library quality check using TapeStation (10) Run library in the NextSeq Illumina platform. **(B)** Bioinformatic pipeline utilized for HTS data analysis and virus detection.

Two library preparation protocols; Ribo-Zero Total RNA (Ribo-Zero) (Al Rwahnih et al., 2018; Soltani et al., 2021) and small RNA (siRNA)-based libraries (Chen et al., 2012) were compared utilizing RNA extracts of CiLV-C and CiLV-C2 positive samples. Based on the results (**Supplementary Table S1**), we selected the Ribo-Zero Total RNA protocol. HTS libraries were prepared following Illumina’s recommended instruction using the input amount of 500 ng to 1µg of total RNA. Extracted RNA concentration was measured with Qubit fluorometer (Invitrogen, Carlsbad, CA) and quality was checked using TapeStation (Agilent, Santa Clara, CA) as per manufacturer’s instructions. For the library preparation high-quality RNA samples were selected based on RNA integrity number (RIN) and the TruSeq Stranded Total RNA Library Preparation Plant Kit (Illumina) protocol was followed. During this process, Ribosomal (r) RNA was removed, and depleted RNA was fragmented at 94°C for 8 min. After removing the host rRNA the cDNA library was constructed using the fragmented coding and non-coding RNA with Superscript III at 50°C (ThermoFisher, USA). After first and second strand of cDNA synthesis, the 3′-end adenylation was done (**Figure 1A**). The adenylated cDNA products were ligated using IDT for Illumina TruSeq RNA unique dual indexes adapters (Illumina). The libraries were enriched by PCR following the initial denaturation at 98°C for 30 seconds and 11 cycles of denaturation at 98°C for 10 seconds, annealing at 60°C for 30 seconds, and extension at 72°C for 30 seconds followed by extended elongation at 72°C for 7 minutes and hold at 4°C. The PCR products were clean-up using AMPure XP beads (Beckman Coulter) and eluted using resuspension buffer (Illumina, kit). Prior to pooling, individual libraries were quantified using Qubit flurometer and the quality was tested using TapeStation. Twenty libraries were pooled at 4 µM concentration in a total volume of 5 µL. At the final step, the library pool was denatured and further diluted to 1.8 pM before the run on a NextSeq 550 system in 2 × 75 bp (V2) Hi-output sequencing reagent kits (Illumina, USA) (**Figure 1A**). The images from the instrument were processed using the Illumina software to generate FASTQ sequence data files.

### Analysis of HTS data using bioinformatic tools

Adapter trimming and quality filtering of raw reads were performed with Trimmomatic v. 0.39 (Bolger et al., 2014). Clean reads were mapped to a host reference genome (Sweet Orange RefSeq genome GCF_000317415.1 for citrus samples and *Arabidopsis thaliana* reference genome TAIR10.1 for non-citrus samples) using bowtie2 v. 2.4.2 (Langmead and Salzberg, 2012) and mapped reads were removed from next step analysis. Remaining reads were *de novo* assembled using the Metaviral SPAdes v. 3.15.02 assembler (Antipov et al., 2020) with default parameters. Assembled contigs were further scaffolded using CAP3 (Huang and Madan, 1999), and redundant contigs removed using CDHIT (Fu et al., 2012). The resulting contigs were searched against an in-house database generated from GenBank virus and viroid sequences through BLASTn and BLASTx. Those having hits to the virus database were identified as virus contigs and depth of coverage was estimated using bbmap (Bushnell, 2014). All virus contigs with hits to the same reference virus sequence were pooled and used to estimate coverage of the reference. Cleaned non-host derived reads were masked to eliminate problematic low-complexity sequences (Clarke et al., 2019) and used as input in Pathoscope2 (Hong et al., 2014). Pathoscope2 output was used to construct reference-based assemblies and estimate total mapped reads to the virus database (**Figure 1B**).

### Phylogenetic analyses of complete CiLV-C2H2 genome segments with other cileviruses

To determine the complete genome of CiLV-C2H2-RNA1 and -RNA2 including 5′ and 3′ untranslated regions (UTRs), the RNA-ligase-mediated rapid amplification of cDNA ends (RLM-RACE) kit (ThermoFisher Scientific) was used following the manufacture’s instruction. To obtain the 5′ and 3′-UTR encoding amplicons, the 5′ and 3′ RACE gene-specific outer and inner reverse primers were designed based on CiLV-C2H2-RNA1 and -RNA2 genome sequences. TOPO TA cloning vector (ThermoFisher Scientific) was used to clone the RACE amplicons and at least 5-10 clones were bi-directionally sequenced.

To establish the phylogenetic relationship 29 *Cilevirus* nucleotide sequences were inferred in MEGA11 (Tamura et al., 2021) using the Neighbor-Joining method (Saitou and Nei, 1987) supported by 1,000 bootstrap replicates (next to the branches) (Felsenstein, 1985). The pairwise deletion option was used to construct an optimal condensed tree by removing all ambiguous positions for each sequence pair. The evolutionary distances were computed using the Maximum Composite Likelihood method (Tamura et al., 2004). All the trees were drawn to scale, with branch length measured as the number of base substitutions per site (shown next to branch nodes).

## Results

### Identification of cileviruses in single and mixed infection in citrus, swinglea and hibiscus plants using real time RT-PCR assays

**Figure 2:**
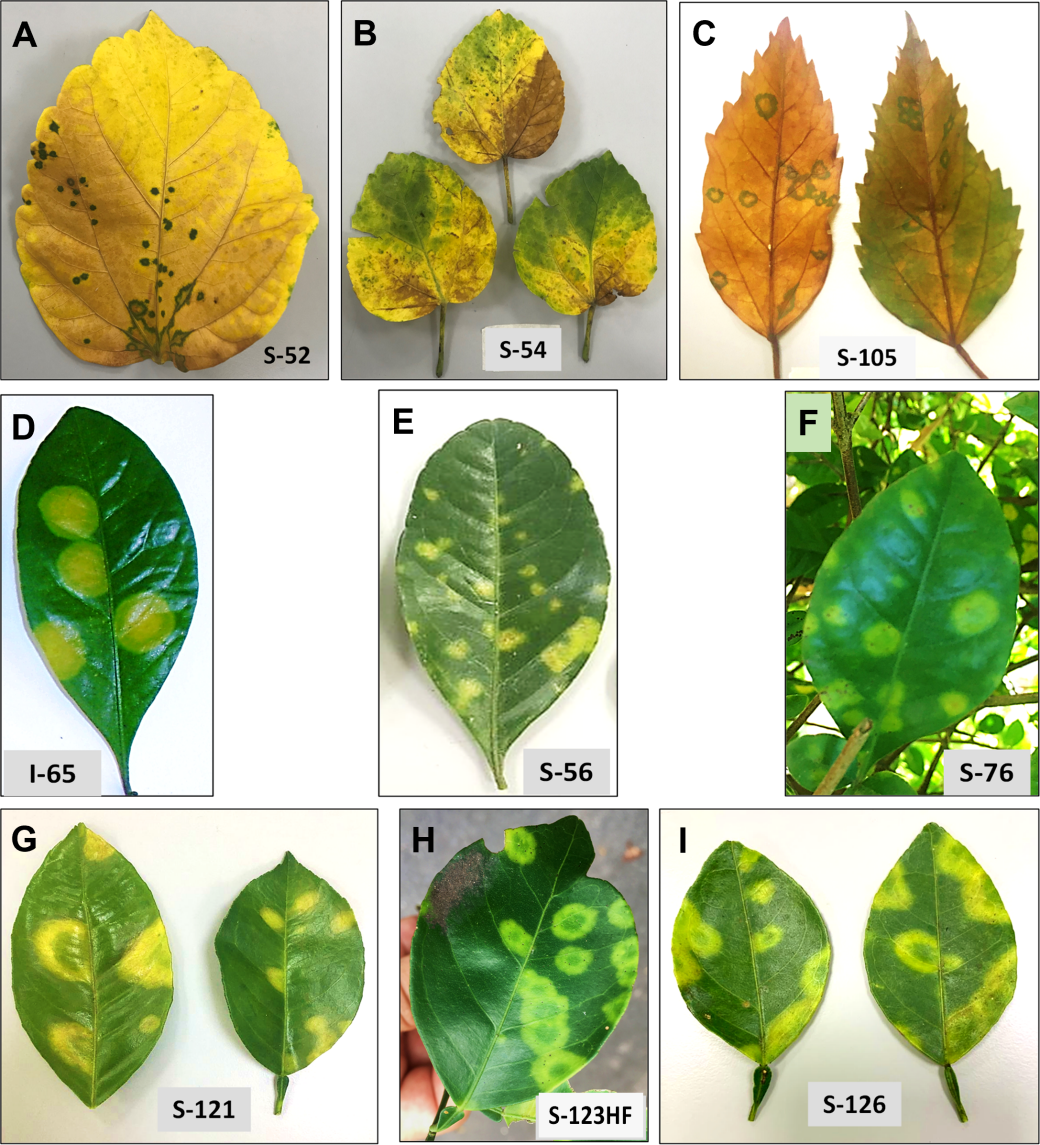
Disease symptoms are associated with single infection of citrus leprosis cytoplasmic 2 (CiLV-C2), and hibiscus strain of CiLV-C2 (CiLV-C2H), respectively, in *Hibiscus rosa-sinesis* **(A, B)**, *Swinglea glutinosa* spp. **(D, E)** and *Citrus sinensis* **(G, H)** and with mixed infection of CiLV-C2 and CiLV-C2H in *H. rosa-sinesis* **(C)**, *S. glutinosa* **(F)** and *C. sinensis* **(I)**.

Our study is primarily focused on the utilization of HTS for the accurate diagnosis and identification of cytoplasmic- and nuclear-types of CiLVs those may not be detected using traditional PCR-based tools. Towards that goal, the leaves of *Citrus* spp., *S. glutinosa*, and *H. rosa-sinensis* with BTV-like symptoms (**Figure 2**) were collected from multiple locations in Colombia (**Table 1**). In total, 20 BTV-like samples from three different hosts were tested to confirm the presence of *Cilevirus* and *Dichorhavirus* infections using species-specific real-time RT-PCR (Choudhary et al., 2015; Adducci et al., 2018, Roy et al., 2022). All the citrus, swinglea, and hibiscus samples associated with BTV-like disease symptoms were negative for *Dichorhavirus* but most of them (18/20) were positive for cileviruses CiLV-C2 or CiLV-C2H or both (**Table 1**). None of the samples were positive to CiLV-C. The concentrations of each virus in the individual plant species were tentatively determined based on their Ct value (**Table 1**). Out of 8 citrus isolates tested, 2 isolates were negative, and three were identified having mixed infection with CiLV-C2 and -C2H whereas all other isolates were positive to CiLV-C2 or -C2H. Single infection of CiLV-C2 and -C2H was identified in one and three of the 6 hibiscus isolates, respectively, whereas mixed infection identified in one isolate and one tested negative. Only one CiLV-C2 infected swinglea isolate was identified, while the other 5 samples were positive to CiLV-C2H either in single or mixed infection with CiLV-C2. Single infections of CiLV-C2 and CiLV-C2H were detected in *Citrus* spp., swinglea and hibiscus, producing Ct values in between 20-24 (S-121 and S-123HF), 14-21 (S-56 and I-65) and 24-27 (S-54 and S-52), respectively (**Table 1**). The isolates named in the brackets were chosen for downstream HTS assay. Citrus isolate S-19, S-125 and S-126, swinglea isolate S-76 and S-191 and hibiscus isolate S-105 were identified as mixed isolates with CiLV-C2 and -C2H infection (**Table 1**). Concentrations of CiLV-C2 and -C2H in mixed infections varied based on random sampling of the host species, as the Ct value of CiLV-C2 and -C2H ranges from 13-28 and 17-30, respectively. All the single CiLV-infected RNA templates were further diluted (Ct 22-24) for downstream HTS protocol optimization and stored in -80°C but no further dilution was made for mixed infected RNA extracts.

**Table 1:** Detection of single and mixed infection of citrus leprosis virus -C (CiLV-C), citrus leprosis virus C2 (CiLV-C2), and hibiscus strain of citrus leprosis virus C2 (CiLV-C2H) in total RNA extracts of C*itrus sinensis*, *Swinglea glutinosa* and *Hibiscus rosa-sinensis* using Real time RT-PCR.

				Geographic location in Colombia		Primers and Taqman Probe in Real time RT-PCR				Type of infection			
Host	Species	Family	Sample ID (Isolate Name)	Department	Municipality	Citrus leprosis cileviruses			Plant gene	Single		Mixed	Negative to
						CiLV-C	CiLV-C2	CiLV-C2H	Nad5	CiLV-C2	CiLV-C2H	(CiLV-C2/C2H)	CiLV-C2 & C2H
						Cycle threshold (Ct) value							
Non-citrus												
Hibiscus	*H. rosa-sinensis*	Malvaceae	MHV (S-52)	Meta	Villavicencio	UD^#^	26.71	UD	35.92	YES	NO	NO	NO
			VHFR (S-54)	Valle del Cauca	Andalucia	UD	UD	24.51	35.65	NO	YES	NO	NO
			THM(S-105)	Tolima	Chicoral	UD	17.82	23.94	29.9	NO	NO	YES	NO
			MGrH (S-183)	Meta	Granada	UD	UD	21.32	32.63	NO	YES	NO	NO
			VGH (S-197)	Valle del Cauca	Ginebra	UD	UD	25.77	28.6	YES	NO	NO	NO
			CYH (S-233)	Casanare	Yopal	UD	UD	UD	28.38	NO	NO	NO	YES
Swinglea	*S. glutinosa*	Rutaceace	SW-PC (I-65)	Valle del Cauca	Andalucia	UD	20.52	UD	27.11	YES	NO	NO	NO
			VSAN (S-56)	Casanare	Yopal	UD	UD	13.77	35.66	NO	YES	NO	NO
			VSR (S-74)	Valle del Cauca	Rozo	UD	UD	19.21	25.61	NO	YES	NO	NO
			VSLP (S-76)	Valle del Cauca	Palmira	UD	22.57	22.98	24.66	NO	NO	YES	NO
			TSw (S-191)	Tolima	Ibague	UD	13.18	17.03	19.16	NO	NO	YES	NO
			VGSw (S-201)	Valle del Cauca	Ginebra	UD	UD	12.54	17.46	NO	YES	NO	NO
Citrus												
Sweet orange	*Citrus sinensis*	Rutaceace	MGNV-3 (S-19)	Meta	Guamal	UD	27.65	29.99	21.93	NO	NO	YES	NO
			MVNC1 (S-121)	Meta	Villavicencio	UD	19.97	UD	22.11	YES	NO	NO	NO
			MRNC (S-123HF)	Meta	Restrepo	UD	UD	24.08	26.78	NO	YES	NO	NO
			MRNV (S-126)	Meta	Restrepo	UD	19.75	28.31	25.99	NO	NO	YES	NO
Mandarin	*C. reticulata*		MY002 (S-22)	Casanare	Yopal	UD	20.87	UD	23.92	YES	NO	NO	NO
			MRMC (S-125)	Meta	Restrepo	UD	21.75	26.46	26.03	NO	NO	YES	NO
			MGrM (S-236)	Meta	Granada	UD	UD	UD	18.64	NO	NO	NO	YES
Rough lemon	*Citrus* x *limonia*		VLRPC (S-71)	Valle del Cauca	Pradera	UD	UD	UD	30.82	NO	NO	NO	YES

#'UD' Undertermined

### Symptoms of cileviruses in citrus, swinglea and hibiscus plants in single and mixed infection

*S. glutinosa* and *H. rosa-sinensis* are perennial ornamental plants belonging to the Rutaceae and Malvaceae families, respectively. BTV symptoms vary among these plant species. Chlorotic spots and ringspots of varied sizes on the leaves, like those caused by cileviruses was observed on sweet orange, mandarin and swinglea leaves. Natural infection by CiLV-C2 and CiLV-C2H produced green ringspots with internal chlorotic spots in senescing *H. rosa-sinensis* leaves. Chlorotic round lesions with green borders were also observed in the sample S-105 infected with both CiLV-C2 and CiLV-C2H (**Figure 2**).

### Evaluation of HTS protocols for detection of cileviruses in citrus, swinglea and hibiscus

To initiate the evaluation of HTS methodology, the positive RNA extracts of CiLV-C and CiLV-C2 samples were detected using real-time PCR (data not shown). For virus detection, a HTS library preparation methodology was selected by comparing the data generated by the Ribo-Zero total RNA and siRNA library preparation methods. Comparison of Ribo-Zero total RNA and siRNA deep sequencing results revealed that both methods provided almost 100% coverage of the RNA1 and RNA2 segments of the CiLV-C and CiLV-C2 genomes. Furthermore, both libraries also contained viroid sequences. Greater viroid sequence coverage was found in the Ribo-Zero library compared with the siRNA library (**Supplementary Table 1**). Following this Ribo-Zero protocol, we have generated three libraries from each host (citrus, swinglea and hibiscus), representing single as well as mixed infections with RNA extracts of CiLV-C2 and CiLV-C2H. A total of nine libraries were sequenced and bioinformatic analyses revealed the viruses present within each host plant.

### HTS data analysis: Single infection of CiLV-C2 in hibiscus, swinglea and citrus

The analysis of HTS data from hibiscus isolate S-52 revealed CiLV-C2-RNA1 and -RNA2 genome sequences. Both segments had ~98% coverage and shared 99.26% and 95.77% nt sequence identity with the reference CiLV-C2-RNA1 and -RNA2 genome sequences (Acc. No. JX000024-25) (**Table 2**). In addition, the HTS data analysis of S-52 hibiscus library also revealed the presence of hibiscus chlorotic ringspot virus (HCRSV) with genome coverage of 99.95%, which was present in the sample used as the CiLV-C2 positive RNA template. Similarly, 99.83% of RNA1 and 96.35% of RNA2 sequences of CiLV-C2 genome were detected in swinglea isolate I-65, which shared 98.86% and 94.87% nt sequence identity with CiLV-C2-RNA1 and -RNA2 genome segments (Acc. No. JX000024-25), respectively. Bioinformatic analysis of HTS data of citrus isolate S-121 generated 2-3 contigs for CiLV-C2-RNA1 and -RNA2 segments with genome coverage of 97.95% and 98.96%, respectively. Although other cileviruses were not detected in this library, isolate S-121 had mixed infection with citrus tristeza virus (CTV). The HTS data analysis confirmed the presence of single infection of CiLV-C2 in hibiscus (S-52), swinglea (S-54) and *C. sinensis* (S-121) isolates (**Table 2**).

**Table 2:** List of plant samples used for High Throughput Sequencing and bioinformatic analysis.

Host	Isolates Name	Raw Reads	Viral Hit		Genome Coverage	Percent NT identity	Depth of Coverage	Number of Contigs	Sequences Aligned with Viruses	Virus Detected
			Accession number	Length						
Hibiscus	S-52	27,260,461	JX000024	8717	97.66	99.26	1079.86	1	Citrus leprosis virus C2 isolate L147V1 segment RNA1	CiLV-C2-RNA1 & RNA2, Hibiscus chlorotic ringspot virus, and Hibiscus latent Fort Pierce virus
			JX000025	4989	98.26	95.77	3095.99	2	Citrus leprosis virus C2 isolate L147V1 segment RNA2	
			MK279671	3945	99.95	97.46	49012.62	1	Hibiscus chlorotic ringspot virus isolate SBO1	
	S-54	13,272,329	MK279671	3945	99.95	95.68	2243.28	1	Hibiscus chlorotic ringspot virus isolate SBO1	CiLV-C2H-RNA1 & -RNA2, Hibiscus chlorotic ring spot virus
			KC626783	8725	99.60	97.38	890.80	1	Citrus leprosis virus cytoplasmic type 2 isolate hibiscus segment RNA1,	
			KC626784	5019	85.57	90.92	2227.33	1	Citrus leprosis virus cytoplasmic type 2 isolate hibiscus segment RNA2	
	S-105	25,084,215	JX000024	8717	99.20	98.98	14041.50	22	Citrus leprosis virus C2 isolate L147V1 segment RNA1	CiLV-C2H-RNA1 & -RNA2 and CiLV-C2-RNA1
			KP828049	6453	98.85	99.05	154427.32	1	Hibiscus latent Fort Pierce virus isolate HLFPV-BR	
			KY933060	3908	100.00	97.33	78013.62	1	Hibiscus chlorotic ringspot virus isolate XM	
			MG253804	4991	65.12	89.48	8677.31	3	Hibiscus-infecting cilevirus isolate HiCV segment RNA2	
			KC626783	8725	38.05	97.18	1355.02	11	Citrus leprosis virus cytoplasmic type 2 isolate hibiscus segment RNA1	
			KC626784	5019	14.05	91.77	15218.15	4	Citrus leprosis virus cytoplasmic type 2 isolate hibiscus segment RNA2	
Swinglea	I-65	26,995,264	JX000024	8717	99.83	98.86	9632.64	1	Citrus leprosis virus C2 isolate L147V1 segment RNA1	CiLV-C2-RNA1 & -RNA2
			JX000025	4989	96.35	94.87	8570.99	6	Citrus leprosis virus C2 isolate L147V1 segment RNA2	
			KF800045	6663	34.16	76.95	23.40	2	Citrus endogenous pararetrovirus clone CB17-3	
	S-56	27,599,561	KC626783	8725	99.94	97.39	40499.65	1	Citrus leprosis virus cytoplasmic type 2 isolate hibiscus segment RNA1	CiLV-C2-RNA1 & -RNA2
			KC626784	5019	85.24	93.86	57038.36	2	Citrus leprosis virus cytoplasmic type 2 isolate hibiscus segment RNA2	
	S-76	18,861,986	JX000024	8717	99.37	99.32	627.90	1	Citrus leprosis virus C2 isolate L147V1 segment RNA1	CiLV-C2-RNA1 & -RNA2, CiLV-C2H-RNA1 & RNA2, and Endogenous pararetro virus
			JX000025	4989	98.90	91.18	2759.54	1	Citrus leprosis virus C2 isolate L147V1 segment RNA2	
			KC626783	8725	88.54	98.37	576.60	5	Citrus leprosis virus cytoplasmic type 2 isolate hibiscus segment RNA1	
			KY609920	4089	86.38	81.43	954.63	12	Citrus endogenous pararetrovirus strain SPBR01	
			KC626784	5019	51.90	97.07	1753.64	4	Citrus leprosis virus cytoplasmic type 2 isolate hibiscus segment RNA2	
Citrus	S-121	15,910,683	JX000024	8717	99.61	99.38	154.46	2	Citrus leprosis virus C2 isolate L147V1 segment RNA1	CiLV-C2-RNA1, & -RNA2
			JX000025	4989	96.05	91.20	107.25	3	Citrus leprosis virus C2 isolate L147V1 segment RNA2	
	S-123HF	19,380,842	AY285668	409	79.22	98.77	178.98	1	Citrus tristeza virus isolate BAN-2 (T3k17) ORF1a	CiLV-C2H-RNA1 & -RNA2 and CiLV-C2-RNA22
			JX000025	4989	99.64	96.48	4894.93	4	Citrus leprosis virus C2 isolate L147V1 segment RNA2	
			KC626783	8717	99.90	88.77	2922.77	1	Citrus leprosis virus cytoplasmic type 2 isolate hibiscus segment RNA1	
			KY609920	4089	51.16	92.96	664.32	19	Citrus endogenous pararetrovirus strain SPBR01	
			KC626784	5019	91.79	89.62	2299.09	2	Citrus leprosis virus cytoplasmic type 2 isolate hibiscus segment RNA2	
	S-126	9,743,431	JX000024	8717	100.00	98.90	1372.17	1	Citrus leprosis virus C2 isolate L147V1 segment RNA1	CiLV-C2-RNA1, & -RNA2, CiLV-C2H-RNA1
			JX000025	4989	95.07	97.52	2680.99	4	Citrus leprosis virus C2 isolate L147V1 segment RNA2	
			KC626783	8725	16.81	97.22	55.39	4	Citrus leprosis virus cytoplasmic type 2 isolate hibiscus segment RNA1	

Summary of viral assemblies confirm the single and mixed infection of cileviruses in citrus, swinglea and hibiscus and the recovery of RNA1 and RNA2 genome sequences of *Cilevirus colombiaense* (citrus leprosis virus C2, CiLV-C2) and its hibiscus strain (CiLV-C2H) with known virus sequences, in the NCBI GenBank.

### HTS data analysis: Single infection of CiLV-C2H in hibiscus, swinglea and citrus

In the process of optimization, next we utilized the HTS protocol for identification of CiLV-C2H in hibiscus, swinglea and citrus. The HTS analysis of hibiscus isolate S-54 library revealed the presence of the CiLV-C2H-RNA1 and RNA2 sequences with 99.60%, and 85.57% genome coverage, which shared 97.38%, and 90.92% nt sequence identity to the reference sequence in the GenBank (Acc. No. KC626783-84). The presence of 99.95% of the genome of HCRSV in the CiLV-C2H HTS library reveals the presence of HCRSV in mixed infection with cileviruses, which is common in hibiscus in Colombia (**Table 2**). Bioinformatic analysis showed coverage of 99.94% of CiLV-C2H-RNA1 and 85.24% RNA2 genome sequences using the HTS data generated from the swinglea isolate S-56 library, which shared 97.39% and 93.86% nucleotide sequence identities with Hawaiian CiLV-C2H isolate sequence (Acc. No. KC626783-84). The citrus isolate S-123HF contained CiLV-C2H-RNA1 and -RNA2 with ~99.90%, and 91.79% genome coverage and 88.77%, and 89.62% nt identity to the CiLV-C2H reference sequence (KC626783-84), in mixed infection with poorly covered partial CTV sequence (**Table 2**). Interestingly, 4 contigs of -RNA2 with coverage of 99.64% of its genome were also identified with more than 96% nucleotide sequence identity with CiLV-C2-RNA2 (Acc. No. JX000025). The current real time PCR assay specific for CiLV-C2-RNA1 failed to detect CiLV-C2 sequences in this mixed infection with CiLV-C2H in Citrus isolate S-123HF but HTS technology did identify the sequences irrespective of any RNA segments of CiLV-C2 or CiLV-C2H genome (**Table 3**).

**Table 3:** Confirmatory diagnosis of real time RT-PCR positive citrus leprosis virus C2 (CiLV-C2) and its hibiscus strain CiLV-C2H in hibiscus, citrus, and swinglea plants in Colombia using analysis of High Throughput Sequencing (HTS) data.

Host	Sample ID	Methods tested for detection of cileviruses in single and mixed infection using				Determined type of infection using	
		Real Time PCR (Ct value)		High Throughput Sequencing (HTS)			
		CiLV-C2	CiLV-C2H	CiLV-C2	CiLV-C2H	Real Time PCR	HTS
**Hibiscus**	S-52	27.00	UD	RNA-1 and RNA-2	None	Single	Single
	S-54	UD	22.00	None	RNA-1 and RNA-2	Single	Single
	S-105	18.00	24.00	RNA-1 but No RNA-2	RNA-1 and RNA-2	Mixed	Mixed
**Citrus**	S-121	21.00	UD	RNA-1 and RNA-2	None	Single	Single
	S-123HF	UD	24.00	RNA-2 but No RNA-1	RNA-1 and RNA-2	Single	Mixed
	S-126	19.00	28.00	RNA-1 and RNA-2	RNA-1 but No RNA-2	Mixed	Mixed
**Swinglea**	I-65	18.00	UD	RNA-1 and RNA-2	None	Single	Single
	S-56	UD	13.00	None	RNA-1 and RNA-2	Single	Single
	S-76	25.00	25.00	RNA-1 and RNA-2	RNA-1 and RNA-2	Mixed	Mixed

UD, Undetermined.

### HTS data analysis: Mixed infection of CiLV-C2 and CiLV-C2H in hibiscus, swinglea and citrus

Preliminary real time RT-PCR data based on cycle threshold (Ct) values identified samples (6/20) doubly infected with CiLV-C2 and CiLV-C2H. The HTS assay followed by bioinformatic analysis was utilized to identify both CiLV-C2 and CiLV-C2H in the same library (**Figure 1B**). We selected the citrus isolate S-126, swinglea isolate S-76 and hibiscus isolate S-105 to evaluate the HTS library preparation protocol. HTS data identified the RNA1 genome sequences of CiLV-C2 and CiLV-C2H in citrus (S-126), swinglea (S-76) and in hibiscus (S-105). Interestingly, both the RNA1 and RNA2 genome sequences of CiLV-C2 and CiLV-C2H were identified only in the swinglea isolate S-76 but RNA2 genome sequences of CiLV-C2 and CiLV-C2H were not found in hibiscus isolate (S-105) or in citrus isolate (S-126), respectively (**Table 3**). Contigs of RNA1 genome segments of the CiLV-C2H and CiLV-C2 generated from citrus (S-126) and hibiscus isolates (S-105) libraries shared 97.22% sequence identity with the CiLV-C2H-RNA1 (Acc. No. KC626783) and 98.98% with CiLV-C2-RNA1 (Acc. No. JX000024), even though the respective virus genome coverage found in citrus and hibiscus isolates were 16.81% and 99.20%. (**Table 2**). To confirm the complete genome sequences of CiLV-C2 and CiLV-C2H in plants with mixed infections, isolate virus/strain specific overlapping RT-PCR assays were used (Roy et al., 2013b, Roy et al., 2018c). Further amplicon sequencing confirms the presence of CiLV-C2 and CiLV-C2H RNA1 genome in citrus, swinglea and hibiscus and absence of RNA2 genome of CiLV-C2 and CiLV -C2H in hibiscus and citrus. No virus sequence was observed in mixed infections of citrus and swinglea whereas ~99% genome coverage of hibiscus latent Fort Pierce virus (HLFPV) and HCRSV were retrieved from the S-105 hibiscus isolates (**Table 2**). Results showed the presence of expected CiLVs in single and mixed infection in all the three hosts. In addition, unexpected citrus and hibiscus viruses were also detected in some Ribo-Zero depleted RNA libraries.

### Discovery of new hibiscus strain of citrus leprosis virus C2 in Colombia

**Figure 3:**
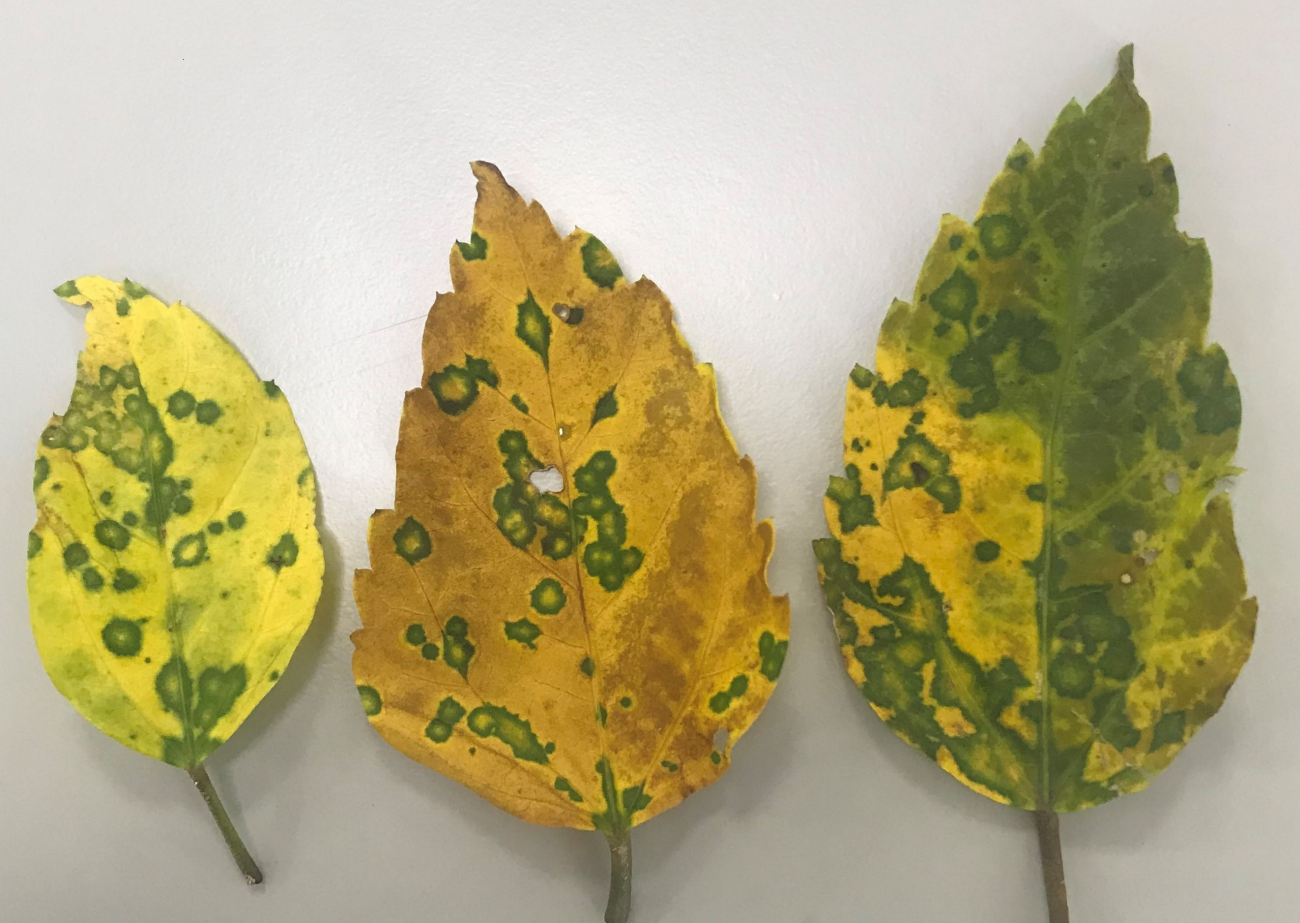
Virus symptom displayed in Hibiscus rosa-sinensis leaves are mixed infected with a new hibiscus strain of citrus leprosis virus C2 (CiLV-C2H2) and hibiscus chlorotic ringspot virus.

*Cilevirus* infection in ornamental hibiscus was previously reported from Hawaii (Melzer et al., 2013) and Florida (Roy et al., 2018a) in the United States and in citrus, swinglea, dieffenbachia and hibiscus in Colombia (Roy et al., 2015a). In 2020, hibiscus samples with green ringspot and green ring lesions with internal chlorotic spots in the senescing leaves were received from Colombia (**Figure 3**). Samples were evaluated using real-time RT-PCR assay for detection of known cile- and dichorha-viruses. Interestingly, the suspected CiL-infected samples from Colombia tested negative for dichorhaviruses and known cileviruses including CiLV-C, CiLV-C2 and CiLV-C2H in real time RT-PCR assays. To identify the pathogen associated with these symptomatic hibiscus leaves, we performed HTS, followed by bioinformatic analysis (as above) that revealed a new hibiscus strain of CiLV-C2. HTS of a Ribo-Zero library of the sample S-38 provided approximately 5 million reads (**Table 4B**). Bioinformatic analysis revealed the presence of a bipartite RNA virus with genome coverage of 99.90% of RNA1 and 98.52% of RNA2 segments. Overall, single contigs of RNA1 and RNA2 genomes shared 88.77% and 90.81% nucleotide identities with sequences of its closest relative CiLV-C2 (JX000024-25). Data analysis also confirmed the presence of a new CiLV-C2 strain (CiLV-C2H2) in hibiscus having mixed infection with HCRSV.

### Genome analysis of CiLV-C2H2 and comparison with CiLV-C2 and CiLV-C2H

Complete genome sequence of hibiscus isolate S-38 was determined after aligning 5′ and 3′ UTR sequences of RNA1 and RNA2 segments with the single contig of each segment identified by HTS. The genome sequence of isolate S-38 reveals the existence of a new hibiscus strain CiLV-C2H2, which is 13,855 nt in length. The RNA1 and RNA2 genomes are composed of 8,745 and 5110 nt, respectively, including the poly(A) tails at the 3´ termini. RNA1 of CiLV-C2H2 has two ORFs and has a similar genome structure with other cileviruses. The start and end positions of the 5´ and 3´ UTRs and each ORFs of RNA1 and RNA2 are shown in **Table 5**. ORF1 of RNA1 encodes a large polyprotein (p285) with conserved domains encoding methyltransferase, protease, helicase, and RNA-dependent RNA polymerase that share 85% nt and 94% amino acid (aa) sequence identity with the known hibiscus strain of CiLV-C2 (Acc. No. KC626783 and MG253805) and 88% nt and 96% aa identity with CiLV-C2 isolate L147V1 infecting citrus (Acc. No. JX000024). ORF2 encodes a putative coat protein gene (p29) and shares 87-90% nt and 94-96% aa sequence identity with Hawaiian hibiscus isolates of CiLV-C2H (Acc. No. KC626783) and CiLV-C2 infecting citrus in Colombia (Acc. No. JX000024), respectively. RNA2 is organized into four ORFs that encode three hypothetical proteins and the conserved movement protein (MP) in the order of 5´-p15-p62-p32(MP)-p24-3´. The intergenic region between ORF1 and 2 of CiLV-C2H2 has 1099 nt of non-coding sequence as compared to 1078 nt found in the genome of the Hawaiian isolate of CiLV-C2H. Interestingly, the Florida hibiscus and Hawaiian passion fruit isolates of CiLV-C2H (Acc. No. MG253804 and MW413438), and L147V1 isolate of CiLV-C2 (Acc. No. JX000025) have a potential extra ORF between ORF1 and ORF2 (described for CiLV-C2H2) that encodes protein p9 or p7, respectively. The most variable region of cileviruses lies between ORF1 and the start codon of hypothetical protein p61. ORF1 of CiLV-C2H2 encodes the hypothetical protein p15 shared 90% nt and 87% aa identity with CiLV-C2 followed by 77% nt and aa identity with CiLV-C2H. Two other hypothetical proteins of CiLV-C2H2-RNA2, p62, and p24, share 92-93% nt and 95-98% aa sequence identity with CiLV-C2, respectively. The MP of CiLV-C2H2 shared 94% nt identity with CiLV-C2 and only 83% nt identity with CiLV-C2H. The maximum sequence divergence was observed between CiLV-C2 and CiLV-C2H2 within the intergenic region (IGR) between ORF1 and ORF2. Interestingly, IGR of CiLV-C2H2 shares only 77% nt identity with CiLV-C2 (Acc. No. JX000025), and 73% nt sequence identities with the CiLV-C2H (Acc. No. KC626784). Overall, the CiLV-C2H2 -RNA1 and -RNA2 shared 88% and 89% nt sequence identities, respectively, with the sequences of CiLV-C2 infecting citrus as compared to the 80-85% nt identities shared with genome segments of the known hibiscus strain of CiLV-C2 (CiLV-C2H) (**Table 4**).

Table 4Application of optimized High Throughput Sequencing protocol: (A) detection of (i) *Cilevirus* genome sequences in Hibiscus in Florida, Passionfruit in Hawaii, and Dieffenbachia in Colombia and (ii) *Dichorhavirus* genome sequences in *Citrus reticulata* in Hawaii and in *Smilax auriculata* in Florida either in single and mixed infection with other citrus, orchid, hibiscus, and watermelon infecting viruses. (B) discovery of a new hibiscus strain of citrus leprosis virus C2 (CiLV-C2H2) in hibiscus in Colombia in mixed infection with hibiscus chlorotic ringspot virus.

**Table 4A:** 

Hosts	Isolates Name	Raw Reads	Viral Hit		Genome Coverage	Percent NT Identity	Depth of Coverage	Number of Contigs	Sequences Aligned with Viruses	Virus Detected
			Accession Number	Length						
Citrus	Hawaii	119,597,157	AB244417	6413	99.98	98.69	10804.94	1	Orchid fleck virus segment RNA 1	OFV-RNA1 & -RNA2, CTV, CEPRV, HLFRV
			AB244418	6001	99.87	98.67	19194.61	1	Orchid fleck virus segment RNA 2	
			AY652909	879	69.74	94.94	1087.83	1	Citrus tristeza virus, p349 gene	
			HF679486	5983	99.03	99.12	72.00	1	Citrus vein enation virus, isolate VE-1	
			KF800045	6663	53.68	88.94	18.63	17	Citrus endogenous pararetrovirus-CB17-3	
			KP828049	6453	58.04	99.29	4.34	11	Hibiscus latent Fort Pierce virus, HLFPV-Br	
			KY609920	4089	83.64	91.34	28.30	20	Citrus endogenous pararetrovirus -SPBR01	
			MH558666	19250	77.95	95.95	10612.21	3	Citrus tristeza virus, islate CN-RB-L13	
			MH593380	19249	55.28	91.98	3590.96	3	Citrus tristeza virus, Isolate CT91-A1	
Hibiscus	Florida	118,206,565	KC626783	8725	85.40	98.83	7.40	10	Citrus leprosis virus cytoplasmic type 2 isolate hibiscus segment RNA1	CiLV-C2H-RNA1 & -RNA2. HLFPV, HCRSV, HLSiV
			KC626784	5019	91.79	89.62	9.66	2	Citrus leprosis virus cytoplasmic type 2 isolate hibiscus segment RNA2	
			KP828049	6453	99.36	98.74	36365.88	3	Hibiscus latent Fort Pierce virus isolate HLFPV-BR	
			KY933060	3908	31.14	95.67	21.69	4	Hibiscus chlorotic ringspot virus isolate XM	
			LC127213	6441	97.33	99.67	26.20	1	Hibiscus latent Singapore virus genomic RNA	
			MK279671	3945	99.87	95.28	25923.60	2	Hibiscus chlorotic ringspot virus isolate SBO1	
Smilax	Florida	28,214,202	AB244417	6413	100.00	98.82	5297.61	1	Orchid fleck virus genomic RNA, segment RNA 1	OFV-RNA1 & RNA2
			AB244418	6001	100.00	98.62	5186.04	1	Orchid fleck virus genomic RNA, segment RNA 2	
Passionfruit	Hawaii	6,522,364	KC626783	8725	89.86	99.28	22.78	10	Citrus leprosis virus cytoplasmic type 2 isolate hibiscus segment RNA1	CiLV-C2H-RNA1 & -RNA2, and WMV
			KC626784	5019	95.12	94.82	26.40	7	Citrus leprosis virus cytoplasmic type 2 isolate hibiscus segment RNA2	
			KX512320	10061	99.55	98.48	177.61	1	Watermelon mosaic virus isolate passiflora	
Dieffenbachia	Colombia - MGD (S-130)	22,919,566	JX000024	8717	96.08	98.55	18.03	15	Citrus leprosis virus C2 isolate L147V1 segment RNA1	CiLV-C2-RNA1 & -RNA2 and CiLV-C2H-RNA1
			JX000025	4989	96.27	99.08	24.65	9	Citrus leprosis virus C2 isolate L147V1 segment RNA2	
			KC626783	8725	26.75	98.48	9.13	11	Citrus leprosis virus cytoplasmic type 2 isolate hibiscus segment RNA1

**Table 4B:** 

Hosts	Isolates Name	Raw Reads	Viral Hit		Genome Coverage	Percent NT Identity				Virus Detected
			Accession Number	Length			Depth of Coverage	Number of Contigs	Sequence Aligned with Viruses	
Hibiscus	Colombia - MLH (S-38)	4,868,027	JX000024	8717	99.90	88.77	6606.08	1	Citrus leprosis virus C2 isolate L147V1 segment RNA1	CiLV-C2H2-RNA1 & -RNA2, and HCRSV,
			JX000025	4989	98.52	90.81	10936.37	1	Citrus leprosis virus C2 isolate L147V1 segment RNA2	
			KY933060	3908	100.00	95.20	16995.54	1	Hibiscus chlorotic ringspot virus isolate XM	

**Table 5:** Percentage of nucleotide and amino acids sequence identities of new hibiscus strain of citrus leprosis virus C2 (CiLV-C2H2) with other cileviruses [citrus leprosis virus C (CiLV-C), citrus leprosis virus C2 (CiLV-C2) and hibiscus strain of CiLV-C2 (CiLV-C2H)] infecting citrus or hibiscus (top panel refer RNA1 and bottom panel refer RNA2).

		Cileviruses					
CiLV-C2H2	Nucleotide Position			CiLV-C		PY_Asu02	PfGSV
		CiLV-C2	CiLV-C2H	CRD	SJP		
**RNA1** Accession Numbers		NC_038848	KC626783	DQ157466	KP336746	MT554532	NC_055653
Entire Genome	1-8745	88/––	85/––	57/––	58/––	58/––	71/––
ORF1 (RdRp)	119-7642	88/96	85/94	59/59	59/59	60/59	72/81
ORF2 (p29)	7710-8504	90/96	87/94	46/32	46/32	47/32	63/63
**RNA2** Accession Numbers		NC_038849	KC626784	DQ157465	KP336747	MT554546	NC_055652
Entire Genome	1-5110	89/––	80/––	46/––	46/––	46/––	59/––
ORF1 (p15)	123-515	90/87	77/77	36/15	35/15	36/15	59/58
Intergenic Region	516-1614	77/––	73/––	39/––	38/––	35/––	39/––
ORF2 (p61)	1615-3249	92/95	83/85	45/30	45/29	46/31	63/60
ORF3 (p32)	3282-4160	94/98	83/91	54/51	55/50	55/50	71/74
ORF4 (p24)	4141-4761	93/98	89/96	61/59	61/60	62/60	78/87

### Phylogenetic analysis

**Figure 4:**
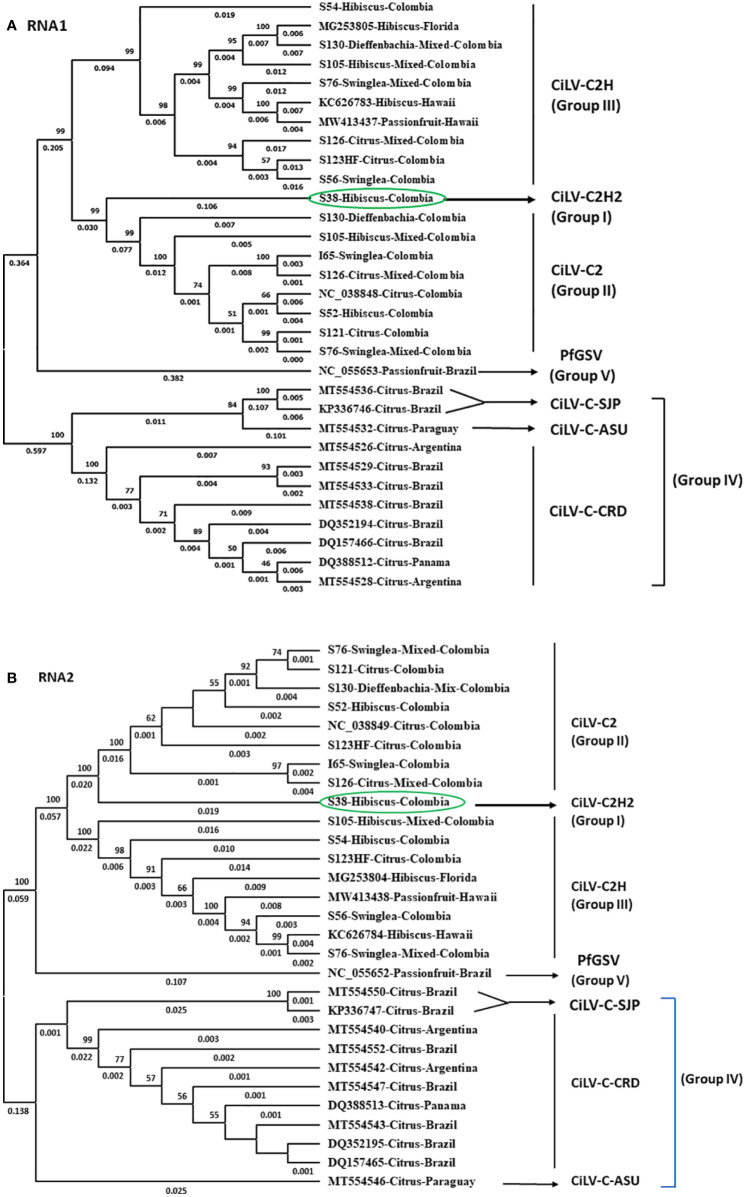
Phylogenetic relationship of new hibiscus strain of CiLV-C2 (CiLV-C2H2) with the members of the nearest *Cilevirus* species; citrus leprosis virus C (CiLV-C), citrus leprosis virus C2 (CiLV-C2), and passion fruit green spot virus (PfGSV) comparing the complete genome sequence of **(A)** RNA1 and **(B)** RNA2 segments.

For the phylogenetic analyses, 16 RNA1 and 14 RNA2 segments of *Cilevirus* genome sequences obtained from this study and 15 *Cilevirus* sequences available in the NCBI GenBank, infecting citrus, hibiscus, swinglea, passionfruit, and dieffenbachia were included. Estimates of evolutionary divergence supported that the hibiscus isolate S-38 was a new hibiscus strain of CiLV-C2 and clustered in all the dendrograms with its nearest relative CiLV-C2 infecting citrus and hibiscus (**Figures 4A, B**). Six more phylogenetic trees were constructed using the amino acid sequences of ORFs from RNA1 (p285 and p29) and RNA2-(p15, p62, p32 and p24) of CiLV-C2H2 and known citrus, hibiscus, and passionfruit *Cilevirus* sequences available in the NCBI GenBank (**Supplementary Figures S1A–F**). The six ORFs of CiLV-C2H2 clustered with CiLV-C2 in all the dendrograms, consistent with it being a second hibiscus strain of the species *Cilevirus colombiaense*. Phylogenetically, CiLV-C2H2 is more closely related to the CiLV-C2 than CiLV-C2H. Based on RNA1 and RNA2 phylogenetic tree analysis, the new hibiscus strain CiLV-C2H2 is placed as the outer member of CiLV-C2 clade but for the evolutionary distance analysis between known cileviruses separated CiLV-C2H2 as only member of group I. The evolutionary distances between CiLV-C2H2 and the other cileviruses were estimated by the bootstrap approach using evolutionary analyses (Tamura et al., 2021). The average nucleotide distances of RNA1 and RNA2 segments between CiLV-C2H2 and the other four groups of cileviruses were analyzed in a pairwise manner (**Table 6**). The average nucleotide distances of RNA1 and RNA2 between CiLV-C2H2 and CiLV-C2H were significantly greater than the distances between CiLV-C2H2 and CiLV-C2. Overall, of the six ORFs, the p15 sequences show maximum evolutionary divergence between CiLV-C2 and CiLV-C2H2 genomes (data not shown).

**Table 6:** Evolutionary divergence of new hibiscus strain of citrus leprosis virus C2 (CiLV-C2H2) with other cileviruses occupied different clades in the phylogenetic trees based on RNA1 (above diagonal) and RNA2 (below diagonal) genome segment sequences.

Group	Cilevirus	Average nucleotide distance ± Standard deviation between groups				
		I	II	III	IV	V
I	CiLV-C2H2		0.196±0.038	0.257±0.050	1.429±0.550	0.712±0.187
II	CiLV-C2	0.039±0.025		0.244±0.047	1.437±0.551	0.712±0.191
III	CiLV-C2H	0.081±0.055	0.084±0.057		1.425±0.544	0.705±0.186
IV	CiLV-C	0.322±0.561	0.317±0.546	0.320±0.551		1.480±0.574
V	PfGSV	0.204±0.220	0.204±0.219	0.204±0.219	0.330±0.616	

CiLV-C, citrus leprosis virus C; CiLV-C2, citrus leprosis virus C2; CiLV-C2H, Known hibiscus strain of CiLV-C2; CiLV-C2H2, new hibiscus strain of CiLV-C2; PfGSV, passion fruit green spot virus.

The number of base substitutions per site between sequences in groups (I-V) is based on the pairwise analysis of 31 RNA1 and 29 RNA2 complete *Cilevirus* genome sequences. Analyses were conducted using the Maximum Composite Likelihood model in MEGA11.

### Detection of cile- and dichorha- viruses in various host tissues received from Colombia and United States

Current HTS methodology was applied to identify Brevipalpus transmitted cile- and dichorha-viruses associated with the CiLD complex in several host plants collected from multiple locations in Colombia, and in Hawaii, and Florida in USA. We identified both CiLV-C2 and CiLV-C2H in mixed infection in *Dieffenbachia* sp. in Colombia, which was a known host for cileviruses. The complete genome sequences of these two viruses were not determined in earlier studies and that is why we included infected dieffenbachia RNA template for HTS assay (**Table 4A**). Both the CiLV-C2-RNA1 and -RNA2 contigs generated from the dieffenbachia library had more than 96% genome coverage and shared >98% nt sequence identity with CiLV-C2 (Accession JX000024-25). Bioinformatic analysis did not detect any CiLV-C2H-RNA2 genome sequences but assembled contigs covered 26.75% of the CiLV-C2H-RNA-1 genome sequence, which shared 97.51% nt identity with Hawaiian CiLV-C2H isolate (**Table 4A**). Overlapping RT-PCR assays were used to bridge the sequence gap and determine the complete genome sequence of CiLV-C2H-RNA1 but failed to amplify any amplicon of RNA2 genome segment using CiLV-C2H-RNA2 specific or CiLV-C2 generic primer pairs described in our previous studies (Roy et al., 2013b; Roy et al., 2018c).

Furthermore, HTS data analysis identified the CiLV-C2H in mixed infection with watermelon mosaic virus in a Hawaiian passion fruit (*P. edulis*) sample received from Honolulu, Hawaii (Courtesy to Dr. Micheal Melzer). Passion fruit isolates of CiLV-C2H shared more than 99% and 94% nt identities with hibiscus isolates of CilV-C2H reported from Hawaii (KC62683-84) (**Table 4A**). Similarly, CiLV-C2H was also detected from a high Ct value real-time PCR positive hibiscus sample received from Florida. Bioinformatic analysis showed 85.40% genome coverage of CiLV-C2H-RNA1 and 91.79% of CiLV-C2H-RNA2, with 98.83% and 89.62% nt identity to the reference Hawaiian isolate sequence in the GenBank (KC62683-84). In the same library, we also identified the HCRSV, HLFPV, and hibiscus latent Singapore virus (**Table 4A**). In addition, current HTS assay also identified the *Dichorhavirus* OFV-Orc strain in rough lemon sample received from Hilo, Hawaii in mixed infection with CTV and citrus vein enation virus. HTS of Greenbrier (*S. auriculata*) samples received from Florida resulted in the identification of OFV-Orc strain. Both the RNA1 and RNA2 contigs of OFV generated from the greenbrier library had 100% genome coverage and shared >98% nt sequence identity with OFV-Orc2 reported in cymbidium orchid in Japan (Accession AB244417-18) (**Table 4A**).

## Discussion

Citrus leprosis is the most economically important BTV disease in the Americas and an emerging threat to US citrus industry. To prevent the establishment of, or to eradicate the virus, accurate virus detection methods are indispensable. Several species of BTVs cause leprosis-like disease symptoms in *Citrus* spp. and it is possible that these symptoms were either caused by or associated with one or more cile- (CiLV-C, CiLV-C2, CiLV-C2H) or dichorha-viruses (CiLV-N, CiCSV, OFV). Traditional serological (Choudhary et al., 2013; Choudhary et al., 2014; Choudhary et al., 2017), and molecular (Roy et al., 2020; Chabi-Jesus et al., 2021) methods were developed earlier for detection of citrus leprosis viruses but these methods may fail to detect new species, or variants of known species of BTVs. The interpretation of diagnostic test results has become more complex since the description of 12 BTVs/strains as part of the CiLD complex. In 2006, HTS was first used in plant virology (Wren et al., 2006) and since then HTS revolutionized plant virus diagnostics by enabling the detection of known viruses as well as the discovery of novel viruses in a variety of plant samples (Villamor et al., 2019). HTS can be utilized to characterize the genome of new species or evolving strains of BTVs associated with the CiLD complex, which will further provide crucial information for identifying diagnostic targets and important insights to enable a more effective CiLD control strategy.

CiL is associated with two unrelated taxa of BTVs (*Cilevirus* and *Dichorhavirus*) and is transmitted by the *Brevipalpus* spp. to a wide range of host plants. To standardize the HTS protocol for detection of viruses associated with regulated CiLD complex, we have chosen three most common plant hosts for detection of cytoplasmic CiLV, belonging to the families, *Rutaceae* (citrus and swinglea) and *Malvaceae* (hibiscus). The extraction of total nucleic acids from plants of the *Rutaceae* using the RNAeasy Plant Mini Kit (Qiagen) is comfortable than from plants belonging to the *Malvaceae*. Irrespective of the RNA isolation method, the quality and quantity of hibiscus total RNA is always inferior as compared to the total RNA extracted from citrus and swinglea leaves. To improve the RNA quality of hibiscus using Qiagen RNeasy kit, an intermediate heating step @ 65°C for 3 minutes was added to the mixture of supernatant flow-through and 96-100% ethanol before transfer to the pink RNeasy mini spin column. Hibiscus plants have a high content of gummy, mucilaginous substances like polysaccharides, secondary metabolites and especially polyphenolics in leaves and roots that can interfere with downstream applications, such as the PCR and HTS library preparation. Our results showed that the Ribo-Zero Total RNA HTS protocol can be applied for the detection of multiple viruses infecting plants which contain phenolics and secondary metabolites. Three cileviruses (CiLV-C, CiLV-C2 and CiLV-C2H) that infect citrus, swinglea and hibiscus (León et al., 2008; Roy et al., 2015a) were preliminary selected for real time RT-PCR assay. Instead of two different virus species from the family *Kitaviridae*, cileviruses CiLV-C2 and its hibiscus strain (CiLV-C2H) were chosen for optimization of HTS protocol. The thought behind this selection was to evaluate the HTS process for the detection of both the virus and its strain associated with CiLD complex. The CiLV-C2H is one of the 12 BTVs associated with CiLD complex and was reported in hibiscus from Hawaii (Melzer et al., 2013) and Florida (Roy et al., 2018a). CiLV-C2H is the only *Cilevirus* reported to date from the US and has the potential to infect citrus and cause CiLD. To identify other potential hosts of BTVs in Florida and California, RNA extracts of symptomatic citrus, swinglea and hibiscus species were selected for HTS optimization based on the Ct value of real time RT-PCR test. RNA selection was performed before library construction. Because rRNA is the most abundant species of RNA in most plant cells, rRNA depletion is commonly used to prepare HTS libraries utilizing RNA virus templates. By removing host rRNA, many non-viral reads can be eliminated to increase the number of reads mapping to viral RNA (Villamor et al., 2019). When HTS is combined with specific bioinformatic tools it can be used for both known virus detection and new virus discovery. Bioinformatic analysis of HTS data may cover a minimal genome length sufficient for virus detection. On the other hand, nearly complete genome coverage not only can detect known viruses but also identify novel virus species or variants of existing species. In this study, analysis of HTS data confirmed the presence of CiLV-C2 and CiLV-C2H either in single or in mixed infection in all three tested plant species but inconsistent with the real time RT-PCR results. The known natural host range of CiLV-C2H was expanded when HTS was applied successfully for detection of CiLV-C2H in passion fruit in Hawaii (Olmedo-Velarde et al., 2022). The HTS protocol also successfully identified OFV orchid strain 2 (OFV-Orc2) (*Dichorhavirus*) in rough lemon and mandarin trees causing citrus leprosis symptoms (Olmedo-Velarde et al., 2021) in Hawaii and in *S. auriculata* showing yellowing and mottling symptoms in Florida (Dey et al., 2022). HTS followed by bioinformatic analysis was also applied to identify the genome sequence of OFV strain associated with the disease symptoms observed in Liriope, Aspidistra, and Ophiopogon in Florida (Roy et al., 2022) and orchids in California (data, not shown).

Suspected BTVs symptomatic samples of citrus, hibiscus, and dieffenbachia were collected from Meta department, Colombia in 2020 and tested using the *Cilevirus* species specific primers. The symptoms of hibiscus isolate (S-38) were somewhat different than known CiLV-C2H symptoms in hibiscus and failed to produce any Ct value below 37 in the real time RT-PCR assay. The HTS protocol was applied to establish possible virus association with the symptomatic hibiscus sample and identified a new hibiscus strain, CiLV-C2H2, having genome sequence variation with CiLV-C2 and CiLV-C2H. The genome sequence of CiLV-C2H2-RNA1 and -RNA2 were determined and compared with CiLV-C2 and CiLV-C2H to establish its taxonomic classification. The CiLV-C2H2 genome is structurally similar to the genomes of bipartite CiLV-C2 and CiLV-C2H cileviruses, share 88-89% nt identity with CiLV-C2-RNA1 and -RNA2 genome, respectively, and is phylogenetically closer to CiLV-C2 than the CiLV-C2H.

A distinct genomic feature was observed in CiLV-C2H2, despite the high amino acid sequence similarity with CiLV-C2, and its hibiscus strains CiLV-C2H. Interestingly, both CiLV-C2 and CiLV-C2H encodes a 7-kDa transmembrane protein (p7) (Roy et al., 2013b; Melzer et al., 2013) but the ORF p7 is absent in the CiLV-C2H2 genome as the similar genome structure was observed in the RNA2 genome segment of CiLV-C (Locali-Fabris et al., 2006). The RNA2 genome sequences between ORF1 (p15) and ORF2 (p61) [with or without p7 ORF] are the most variable region among cileviruses (Ramos-González et al., 2022). Current demarcation criteria for species of the genus *Cilevirus* are based on <85% aa sequence identity for the proteome but there is no cut off value was determined for cilevirus strain identification. Since the genus *Cilevirus* does not have established guidelines for strain demarcation within the species, in the current study taxonomic classification of *Cilevirus* strain was decided based <90% nt sequence identity (**Table 5**) among all the CiLV-C2 relatives.

In this study, HTS was applied for detection and discovery of viruses of regulatory concern associated with the CiLD complex. During study, we identified inconsistencies between real time RT-PCR and analysis of HTS data. The *Cilevirus* species-specific real-time RT-PCR assay confirmed the presence of citrus leprosis associated viruses (CiLV-C2 and CiLV-C2H) in mixed infection in hibiscus (S-105) and citrus (S-126) isolates and single CiLV-C2H infection in citrus isolate S-123HF (**Table 4**). On the other hand, HTS followed by bioinformatic analysis was unable to detect the CiLV-C2-RNA2 and CiLV-C2H-RNA2 genome sequences in hibiscus (S-105) and citrus (S-126) isolates, respectively, and failed to detect CiLV-C2-RNA2 in citrus isolate S-123HF (**Figure 4**). A similar event like S-123HF was observed when HTS failed to detect CiLV-C2H-RNA2 genome sequences in dieffenbachia (isolate S-130) (**Figure 4**). The primers and probes for bipartite CiLV-C2 and CiLV-C2H cileviruses were designed using RNA1 genome sequence data whereas RNA2 variable sequences were utilized for CiLV-C detection. To find out if discrepancies between HTS and real time RT-PCR assays identify a biological reality, at least two sets of *Cilevirus* species-specific primers and probes need to be designed using both RNA1 and RNA2 genome sequences.

Initially we thought that there was an early-stage contamination or cross-contamination when multiplexing several samples in a single sequencing experiment. To prevent the contamination, we followed the guidelines for reliable use of HTS (Lebas et al., 2022; Massart et al., 2022) and repeated the RNA extraction followed by library preparation in a separate laboratory set up. The new dual indexed libraries were constructed in Molecular Plant Pathology Laboratory (MPPL), Beltsville, MD instead of PPCDL, MD using new kits and reagents. Along with single and mixed cilevirus infected hibiscus, swinglea and citrus samples, healthy and cilevirus negative hibiscus and citrus RNA extracts were also included. Furthermore, conventional RT-PCR assays specific to RNA1 and RNA2 genome segments of CiLV-C2 and CiLV-C2H followed by Sanger sequencing (Roy et al., 2013b; Roy et al., 2018c) were performed. The BLASTn analysis confirmed the sequence of CiLV-C2-RNA2 only in the sample S-123HF and the RNA1 sequence of CiLV-C2 and CiLV-C2H in hibiscus (S-105) and citrus (S-126), respectively. Combination of mixed virus sequence detected in the S-126 sample was also identified in the dieffenbachia diagnostic sample (S-130).

Based on the analysis of conventional RT-PCR and HTS results, we hypothesize that both virus species exist in nature in mixed infections by sharing their RNA2 segment with individual variable RNA1 segment or vice-versa. Eventually, single infections of reassorted strains of CiLV-C2 might evolve in any of the hosts. Reassortment occurs in *Dichorhavirus* with segmented genomes by interchanging their complete genome segments giving rise to stable lineages among strains of OFV (Kondo et al., 2017; Roy et al., 2020). OFV has two orchid strains (OFV-Orc1 and OFV-Orc2) and two citrus strains (OFV-Cit1 and OFV-Cit2) and new strains emerged *via* replacement of complete genome segments. The RNA2 genome segment of OFV-Orc1 and OFV-Cit1 was shared with OFV-Orc2 (Kondo et al., 2017) and OFV-Cit2 (Roy et al., 2020), respectively. Further research is needed to confirm this hypothesis and identify a stable lineage among natural reassortment strains of CiLV-C2 infecting citrus, hibiscus, or dieffenbachia.

Even though the HTS methodology was evaluated to identify cileviruses in citrus, swinglea (*Rutaceae*) and hibiscus (*Malvaceae*) but also were successfully applied to detect cileviruses infecting plants belonging to the families; *Araceae* (*Dieffenbachia* sp.), *Passifloraceae* (*P. edulis*) and dichorhaviruses in *Rutaceae* (*C. sinensis*), and *Smilacaceae* (*S. auriculata*). In addition, same HTS protocol was applied to detect (i) OFV (*Dichorhavirus*) in multiple plant species belonging to the families *Asparagaceae* (*Liriope muscari, Ophiopogon* spp. and *Aspidistra elatior*), *Orchidaceae* (cymbidium and dendrobium), and (ii) CiLV-C2 (*Cilevirus*) inside the polyphagous *Brevipalpus* spp. collected from CiLV-C2 positive swinglea leaves (S-76) (data not shown). Furthermore, evaluated HTS protocol was not only detected several viruses in citrus, hibiscus and other plants but also discovered a new hibiscus strain CiLV-C2H2 from a real time RT-PCR negative hibiscus sample. Overall, the use of this HTS method will enhance the surveillance of regulated viruses associated with CiLD complex in multiple hosts by accurate detection of known viruses and identification of novel virus, strain and recombinant if any present in the sample.

## Data availability statement

The data presented in the study are deposited in the NCBI repository (https://www.ncbi.nlm.nih.gov/), BioProject ID PRJNA895362.

## Author contributions

AR and CP designed the experiments. CP optimized the HTS library preparation and sequencing protocol. SN developed the bioinformatic pipeline and assembled virus sequences. AR executed real time RT-PCR experiment and discovered the new hibiscus strain of CiLV-C2. GL collected all the studied samples from Colombia and sent to PPCDL under permit. CP and AR performed laboratory experiments and analyzed the data with the assistance of SN, VM, YR and MN. AR and CP prepared the original draft of the manuscript. All the authors significantly contributed with reviewing and editing the manuscript and agreed to the published version of the manuscript.

## Supplementary material

Supplementary Figure 1
**(A)** Phylogenetic relationships of new hibiscus strain of CiLV-C2 (CiLV-C2H2) with the members of the nearest *Cilevirus* species; citrus leprosis virus C (CiLV-C), citrus leprosis virus C (CiLV-C2), and passion fruit green spot virus (PfGSV) by comparing the amino acid (aa) sequences of **(A)** ORF1 (p285) and **(B)** ORF2 (p29) of RNA1 segment and **(C)** ORF1 (p15), **(D)** ORF2 (p62), **(E)** ORF3 (p32) and **(F)** ORF4 (p24) of RNA2 genome segment.

Supplementary Table 1 Comparison of small RNA (siRNA) and long-RNA (Ribo-Zero Total RNA) HTS data analysis for detection of citrus leprosis virus C (CiLV-C) and Citrus leprosis virus C2 (CiLV-C2).
