# Supplementary figures and images for: High-throughput sequencing application in the detection and discovery of viruses associated with the regulated citrus leprosis disease complex
Source: Front Plant Sci. 2023 Jan 24;13:1058847. doi: 10.3389/fpls.2022.1058847 (PMC9907091; doi:10.3389/fpls.2022.1058847)

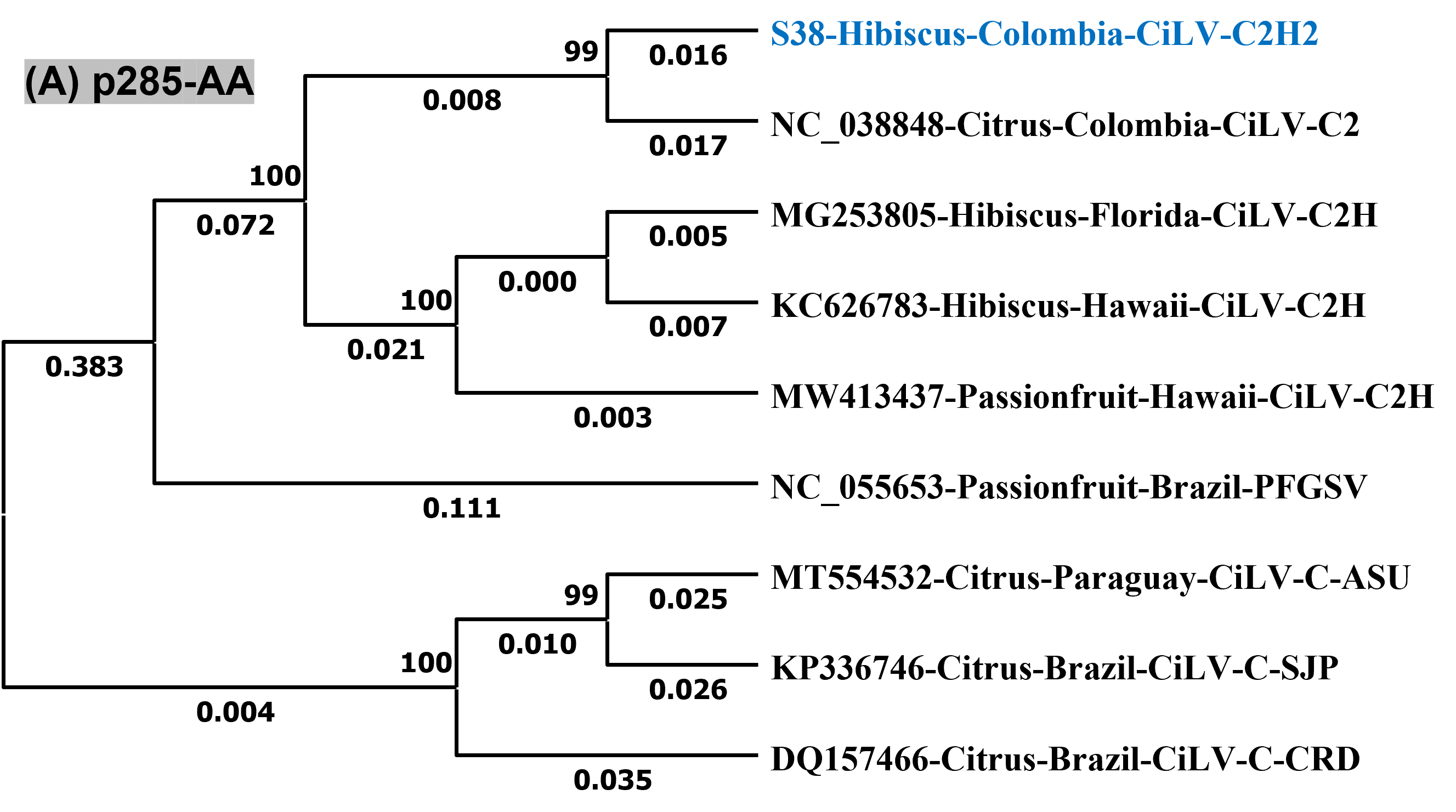

Supplement: Supplementary Figure 1 — (A) Phylogenetic relationships of new hibiscus strain of CiLV-C2 (CiLV-C2H2) with the members of the nearest Cilevirus species; citrus leprosis virus C (CiLV-C), citrus leprosis virus C (CiLV-C2), and passion fruit green spot virus (PfGSV) by comparing the amino acid (aa) sequences of (A) ORF1 (p285) and (B) ORF2 (p29) of RNA1 segment and (C) ORF1 (p15), (D) ORF2 (p62), (E) ORF3 (p32) and (F) ORF4 (p24) of RNA2 genome segment. [file Image_1.tif]

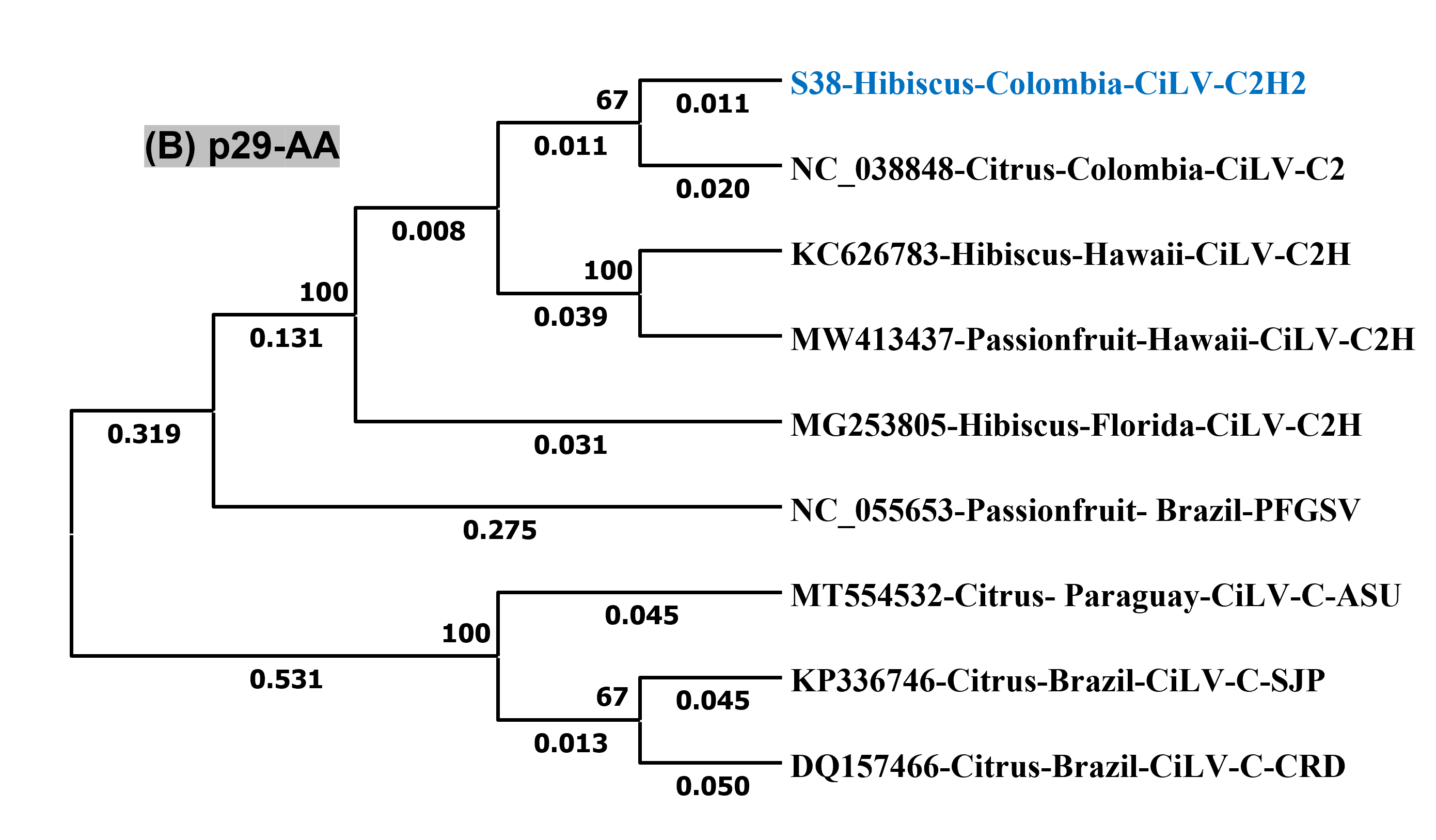

Supplement: Supplementary file 3 [file Image_2.tif]

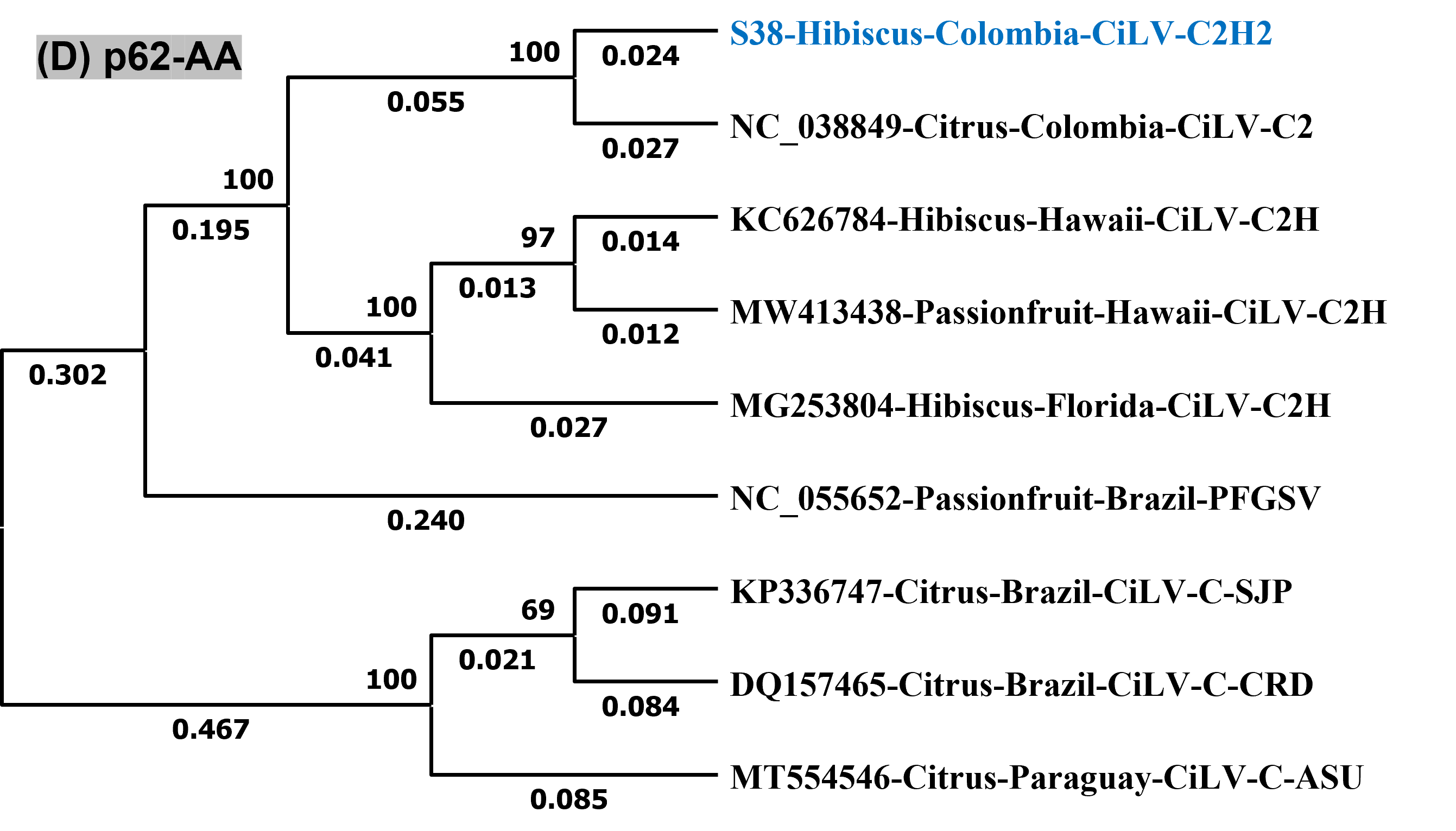

Supplement: Supplementary file 4 [file Image_3.tif]

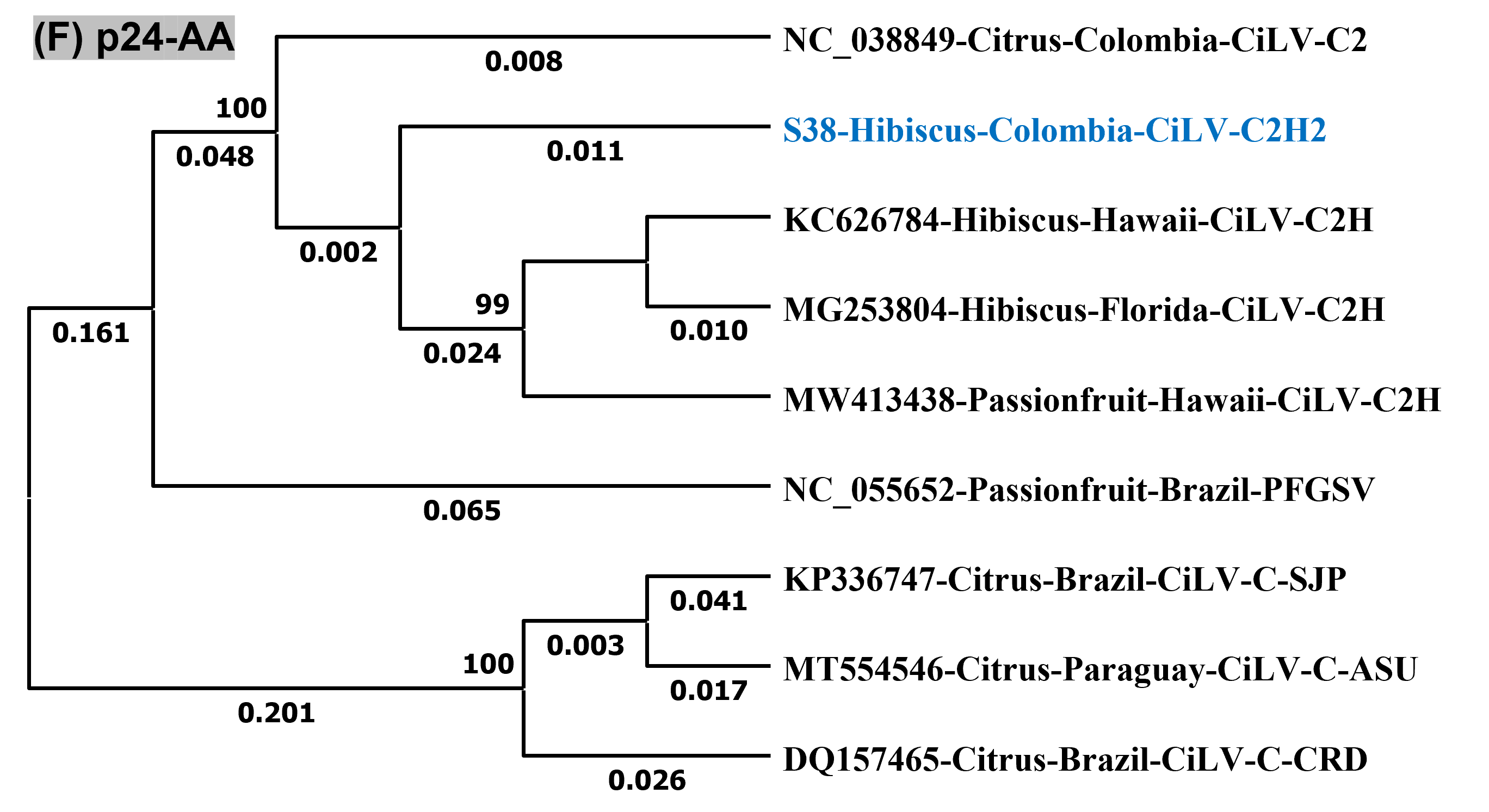

Supplement: Supplementary file 5 [file Image_4.tif]

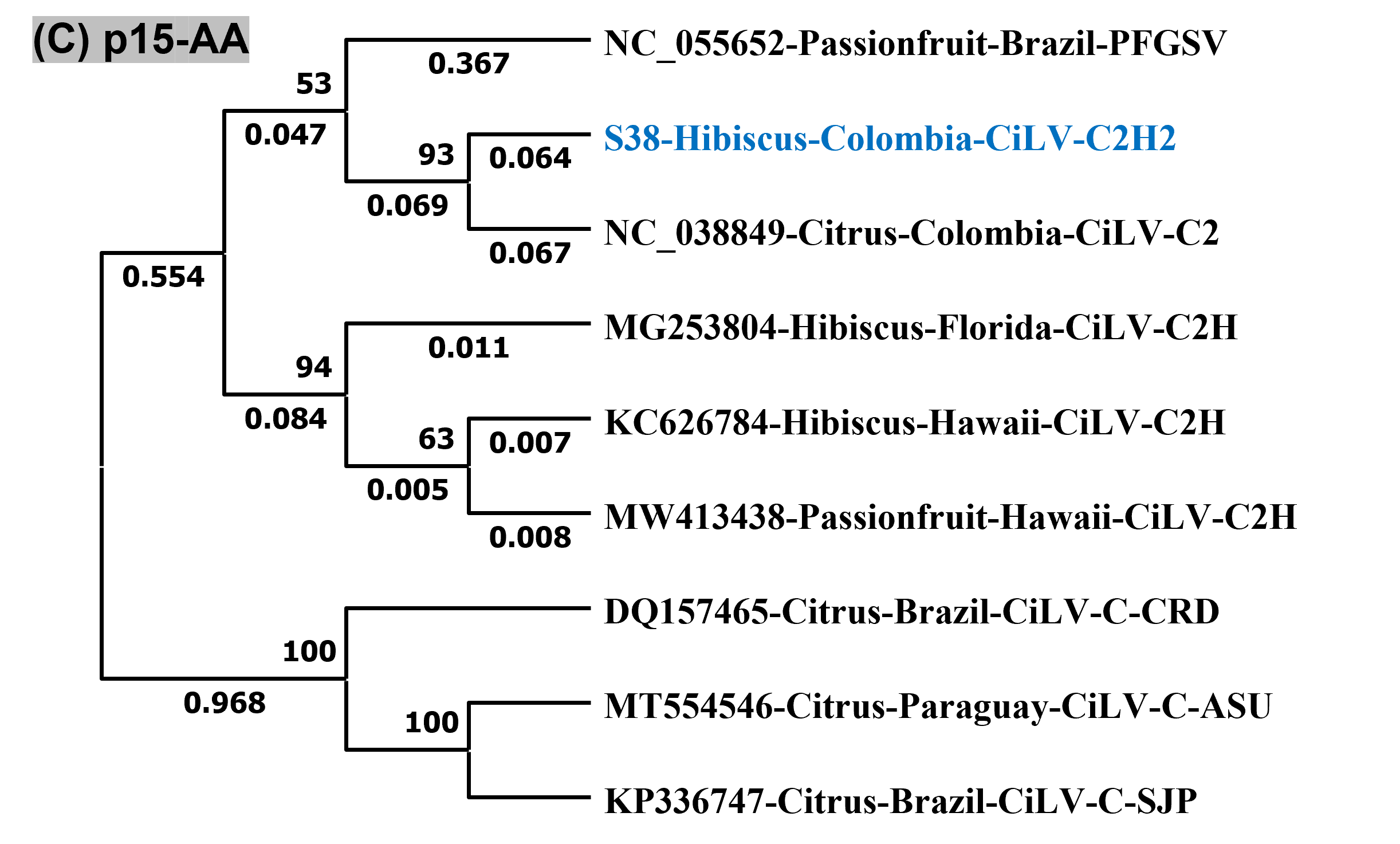

Supplement: Supplementary file 6 [file Image_5.tif]

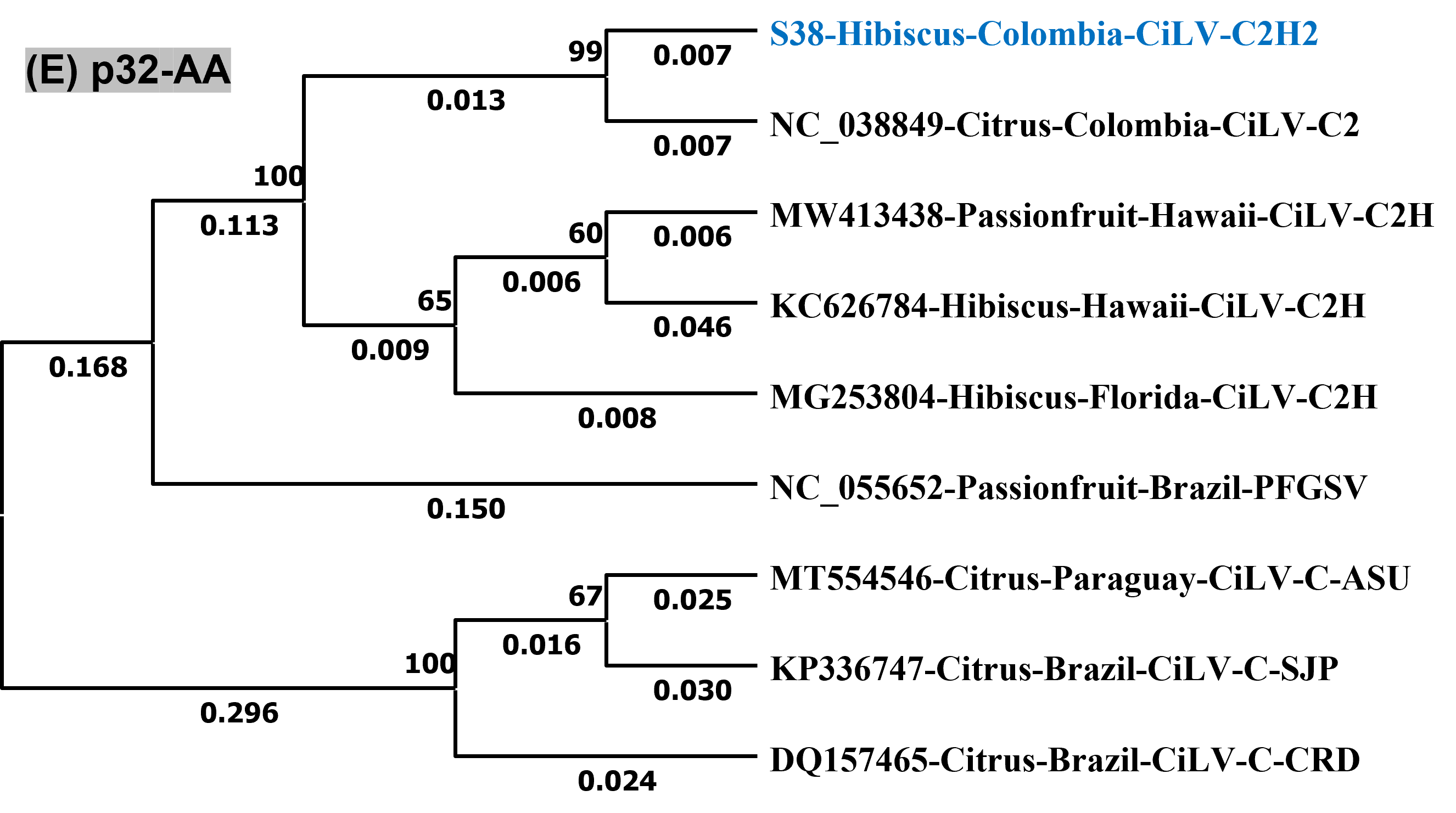

Supplement: Supplementary file 7 [file Image_6.tif]
