# Supplementary material for: High-throughput sequencing application in the detection and discovery of viruses associated with the regulated citrus leprosis disease complex
Source: Front Plant Sci. 2023 Jan 24;13:1058847. doi: 10.3389/fpls.2022.1058847 (PMC9907091; doi:10.3389/fpls.2022.1058847)
Supplement: Supplementary Table 1 — Comparison of small RNA (siRNA) and long-RNA (Ribo-Zero Total RNA) HTS data analysis for detection of citrus leprosis virus C (CiLV-C) and Citrus leprosis virus C2 (CiLV-C2). [file Table_1.docx]

**Supplementary Table 1.** Comparison of small RNA (siRNA) and long-RNA (Ribo-Zero Total RNA) HTS data analysis for detection of citrus leprosis virus C (CiLV-C) and Citrus leprosis virus C2 (CiLV-C2)

| **GenBank ID** | **Virus Code** | **Virus/viroid’s components** | **Genome Coverage (%)** | | **% Identity** | | **Number Mapped Reads** | | **RPM of Total Sample Reads** | |
| --- | --- | --- | --- | --- | --- | --- | --- | --- | --- | --- |
|  |  |  | **siRNA** | **long RNA** | **siRNA** | **long RNA** | **siRNA** | **long RNA** | **siRNA** | **long RNA** |
| DQ352194 | CiLV C | CiLV-C RNA1 | 99.28 | 100 | 98.97 | 98.78 | 214,888 | 38,520,128 | 63,806 | 749,800 |
| DQ352195 |  | CiLV-C RNA2 | 100 | 100 | 99.58 | 99.62 | 96,520 | 14,009,211 | 28,659 | 272,691 |
| EU382205 |  | Citrus viroid III | 97 | 100 | 96.55 | 100 | 146 | 100 | 43 | 2 |
| AY513267 |  | Citrus cachexia viroid | 76 | 100 | 87.96 | 100 | 32 | 401 | 10 | 8 |
| JX000024 | CiLV C2 | CiLV-C2 RNA1 | 100 | 100 | 99.51 | 99.48 | 76,196 | 36,904,824 | 23,287 | 542,936 |
| JX000025 |  | CiLV-C2 RNA2 | 100 | 100 | 99.12 | 99.69 | 82,977 | 25,337,178 | 25,360 | 372,756 |
| JQ911664 |  | Citrus triteza virus CT11A | 100 | 100 | 96.32 | 99.34 | 49,188 | 3,952,309 | 15,033 | 58,146 |
